# Modulation of Antioxidant Capacity, Nutritional Composition, Probiotic Viability After Digestion and Sensory Attributes of Plant-Based Beverages Through Lactic Acid Fermentation

**DOI:** 10.3390/foods14091447

**Published:** 2025-04-22

**Authors:** Matteo Vitali, Mónica Gandía, Guadalupe Garcia-Llatas, Antonio González-Sarrías, Fernando Vallejo, Antonio Cilla, Amparo Gamero

**Affiliations:** 1 Bionutest Research Group—Food Technology Area, Faculty of Pharmacy and Food Sciences, University of Valencia, Av. Vicente Andrés Estellés s/n, 46100 Burjassot, Valencia, Spain; matteo.vitali@uv.es (M.V.); monica.gandia@uv.es (M.G.); amparo.gamero@uv.es (A.G.); 2 Bionutest Research Group—Nutrition and Food Science Area, Faculty of Pharmacy and Food Sciences, University of Valencia, Av. Vicente Andrés Estellés s/n, 46100 Burjassot, Valencia, Spain; guadalupe.garcia@uv.es; 3 Research Group on Quality, Safety and Bioactivity of Plant Foods, Centro de Edafología y Biología Aplicada del Segura, Consejo Superior de Investigaciones Científicas (CEBAS-CSIC), Campus de Espinardo, 30100 Murcia, Spain; agsarrias@cebas.csic.es; 4 Metabolomics Platform, Centro de Edafología y Biología Aplicada del Segura, Consejo Superior de Investigaciones Científicas (CEBAS-CSIC), Campus de Espinardo, 30100 Murcia, Spain; fvallejo@cebas.csic.es

**Keywords:** fermentation, plant-based beverages, probiotics, antioxidant capacity

## Abstract

Fermented plant-based beverages are renowned due to their health benefits and sustainability. This study focuses on developing fermented local beverages from rice, carob, and tiger nuts. The fermentation process with four different commercial starters of lactic acid bacteria was optimized based on pH drop and colony counts at 37 °C and the supplementation with 7.5–15 g glucose/100 mL. Analyses of antioxidant capacity, phytochemical profile, proximate composition and sensory attributes were conducted, along with studies on the gastrointestinal survival of probiotics. Total polyphenols levels and antioxidant capacity followed the order: carob > tiger nut > rice (159.8–218.9 > 34.1–127.9 > 7.2–17.5 mg GAE/L for total polyphenols; 4461.9–15,111.6 > 2916.8–7897.3 > 1845.7–6103.5 µM Trolox/L for ORAC; and 2057.7–4235.3 > 318.9–876.7 > n.d.–239.7 µM Trolox/L for TEAC, respectively). The VEGE061 consortium showed the best results for the majority of parameters analyzed, influencing fat content and fatty acid profiles and increasing monounsaturated fatty acids in tiger nuts while promoting saturated fatty acids in rice beverages. Simulated in vitro digestion significantly reduced probiotic content in tiger nuts, carob, and, to a lesser extent, rice beverages. The beverages showed good sensory attributes, with tiger nut developing lactic and floral notes, carob achieving a balanced aroma profile with VEGE061, and rice displaying pleasant sensory qualities with VEGE033 and VEGE061 consortia. Further research is needed to explore optimal conditions for scaling up the process and strategies to improve probiotic delivery, aiming to increase post-digestion survival. This approach could promote the development of healthy and sustainable food alternatives.

## 1. Introduction

The development of new foods is a growing need in the current world, driven by factors such as population growth, climate change, and the scarcity of natural resources [1,2]. These global challenges require innovative solutions to ensure food security and sustainability. In this context, fermented beverages stand out as a diverse category of foods, recognized for their unique flavors, aromas, and textures, as well as their potential health benefits [3,4]. Among the benefits associated with the consumption of fermented beverages are improved intestinal function, reduced risk of chronic diseases, increased nutrient absorption, and enhanced mood [5]. These benefits are largely due to the presence of probiotics. Lactic acid fermentation is a biochemical process in which carbohydrates, mainly glucose, are transformed into lactic acid by specific bacteria. Through this process, several fermented beverages are produced, and their preservation and nutritional profile are improved. The main bacteria used in lactic acid fermentation throughout history are *Streptococcus thermophilus* and *Lactobacillus bulgaricus*, both recognized as QPS (Qualified Presumption of Safety) [6]. However, there are many other lactic acid bacteria (LAB) with different capacities of adaptation to different substrates and diverse metabolite production. The market for these fermented beverages is steadily growing, driven by the rising demand for healthy foods and the globalization of dietary habits [7,8,9].

Increasing numbers of consumers are seeking food options that are not only delicious but also beneficial to their health. A growing trend is the production of fermented plant-based beverages from local ingredients, which supports the regional economy, reduces the carbon footprint, and promotes a more balanced diet. Ingredients such as tiger nut, carob, and rice, especially in the Valencian Community (Spain), represent appealing options for developing new products due to their economic relevance and local character [4]. This approach can contribute to various Sustainable Development Goals (SDGs), such as SDG2 (Zero Hunger), SDG3 (Good Health and Well-being), SDG12 (Responsible Consumption and Production), and SDG13 (Climate Action) [10]. These Mediterranean ingredients, rich in fiber, vitamins, and minerals, provide valuable nutritional properties and can attract a broad market of consumers. Fermented plant-based beverages made from carob, rice, and tiger nut not only stand out for their nutritional profile but also for their distinctive flavors and aromas, making them products with great market potential [4]. In addition, the antioxidant capacity and phytochemical profile of fermented beverages are of great interest. Antioxidants such as flavonoids, phenols, and tannins present in these beverages could help combat oxidative stress in the body, which can reduce the risk of chronic diseases such as cancer and cardiovascular diseases. Fermentation can enhance the antioxidant capacity by releasing these compounds or decreasing them through catabolic reactions [11,12]. Most existing research focuses on isolated aspects of fermented beverages, such as probiotic survival or bioactive compound content, but rarely integrates analysis of antioxidant capacity, phytochemical profile, proximate composition, and sensory attributes in a comprehensive study [9,10,11,12,13]. There is scarce research on fermented beverages made from rice, tiger nuts, and carob obtained through fermentation by various microorganisms. Moreover, there is also a scarcity of comparative studies that systematically examine how different plant matrices influence the metabolic profiles and viability of lactic acid bacteria [13,14,15]. This knowledge gap limits our understanding of how to optimize these processes for different substrates. Previous studies have demonstrated that the production of fermented beverages is viable using different microorganisms and fermentation processes, although many traditional recipes do not clearly specify fermentation conditions or the microorganisms involved [4,16,17].

Therefore, based on the existing limited research, the aim of this study was to develop three novel and functional plant-based fermented beverages, elaborated with local raw materials such as rice, tiger nut and carob, and evaluate how lactic acid fermentation affects the key properties of the beverages, including antioxidant capacity, bioactive compounds profile, nutritional composition, organoleptic characteristics, and the survival rate of probiotic bacteria after in vitro simulated gastrointestinal digestion. This integrated approach allows us to gain insight into the nutritional, functional and sensory qualities of these fermented products.

## 2. Materials and Methods

### 2.1. Reagents

Reagents used in the fermentation experiments included Mquant pH strips (Merck KGaA, Darmstadt, Germany) and lactose sulphite (LS) agar (BIokar, Paris, France). For fiber assays, a commercial K-TDFR-100A/K kit (Megazyme Ltd., Bray, Co. Wicklow, Ireland) was used. Antioxidant capacity assays included sodium carbonate (Na_2_CO_3_), Folin-Ciocalteu reagent, and gallic acid, all purchased from Sigma Chemical Co. (St. Louis, MO, USA). Other reagents purchased from Sigma Chemical Co. include potassium persulfate (K_2_S_2_O_8_), 2,2′-azino-bis-(3-ethylbenzothiazoline-6-sulfonic acid) (ABTS), 6-hydroxy-2,5,7,8tetramethylchroman-2-carboxylic acid (Trolox) and 2,2′-azobis-(2-amidinopropane) dihydrochloride (AAPH). Ethanol (96%) provided by Scharlau (Barcelona, Spain) was used. Potassium dibasic phosphate (K_2_HPO_4_) and sodium dibasic phosphate (Na_2_HPO_4_) were obtained from Merck (Darmstadt, Germany). Sodium fluorescein was acquired from Fluka Chemie AG (Buchs, Switzerland). Water was purified using a Milli-Q system (Milford, MA, USA). Reagents for the in vitro static digestion assay include human salivary α-amylase (E.C 3.2.1.1), ammonium carbonate, ammonium chloride, anhydrous sodium sulfate, bovine bile, calcium chloride dihydrate, hydrochloric acid with a purity of 37%, magnesium chloride hexahydrate, methanol, pancreatin extract from porcine pancreas, pepsin from the gastric mucosa of pigs (E.C 3.4.23.1), potassium chloride, potassium dihydrogen phosphate, potassium hydroxide, sodium chloride and sodium hydroxide were all purchased from Merck Life Science S.L.U. (Madrid, Spain). Rabbit gastric extract (RGE) was purchased from Lipolytech (Marseille, France). Ethanol, sodium bicarbonate, and sodium hydroxide were obtained from Panreac (Barcelona, Spain).

### 2.2. Beverage Preparation

The raw materials tiger nut (*Cyperus esculentus*), rice (*Oryza sativa*) and carob (*Ceratonia siliqua*) were provided by local farmers. Three types of plant-based beverages were elaborated with these ingredients by a local company (Monvital, Valencia, Spain). The raw materials were chopped and crushed to facilitate pressing and extraction to prepare sifted beverages with the following composition: (i) tiger nut beverage: water (80%) and tiger nut (20%); (ii) carob beverage: water (90%) and carob (10%); and (iii) rice beverage: water (91.9%), rice (8%) and sea salt (0.1%). After manufacturing, the beverages were heat-treated at 85 °C for 5 min. They were then allowed to cool and stored under refrigeration. Once the sample was received at the laboratory, a plate count was performed as a control analysis. The rice and carob beverages showed no evidence of colonies. However, due to microbial load, the tiger nut beverage received an extra heat treatment at 80 °C for 10 min, and no viable colonies were then detected.

### 2.3. Microbial Strains

Four freeze-dried microbial consortia of LAB (Danisco, Denmark), gently donated by Larbus (Madrid, Spain) and suitable for the production of fermented products of plant origin, were used to ferment the beverages. The microbial strains included in each consortium are detailed in Table 1.

### 2.4. Fermentation Process

Fermentations for each type of beverage were carried out in 250 mL flasks containing 150 mL of beverage. The corresponding quantity of inoculum was added according to the manufacturer’s instructions. The flasks were incubated at 30 or 37 °C for 24–72 h to determine the best temperature and time conditions. The pH was measured every 2 h to monitor fermentation, which was considered finished when it reached 4/4.5. To contribute to a more efficient fermentation, different levels of glucose were added: level 1—no glucose addition; level 2—a glucose addition of 7.5 g/100 mL for rice and tiger nut beverages and 15 g/100 mL for the carob beverage; level 3—a glucose addition of 15 g/100 mL for the rice and tiger nut beverage and 30 g/100 mL for the carob beverage.

### 2.5. Microbiological Analysis

Fermented beverages were analyzed to determine the presence of viable bacteria after the fermentation process employing the plate count method (dilutions from 10^−1^ to 10^−6^ in 0.1% peptone water) and LS agar medium. The plates were incubated for 48 h at 37 °C under anaerobic conditions until typical colonies appeared.

### 2.6. Total Soluble Polyphenols and Total Antioxidant Capacity

Due to the absence of a standardized method for measuring total antioxidant capacity (TAC), it is recommended to utilize two or more distinct methods based on different chemical reactions and mechanisms. Ideally, TAC measurement should encompass both hydrogen atom transfer (HAT) and electron transfer (ET). The oxygen radical absorbance capacity (ORAC assay) reflects HAT reactions, while the TSP (total soluble polyphenols Folin−Ciocalteu method) and Trolox equivalent antioxidant capacity (TEAC) assays represent ET reactions. These specific methods were chosen due to their established application in assessing the antioxidant capacity of foods and dietary supplements, as highlighted in a seminal review [18]. The antioxidant capacity was assessed during the fermentation of the three beverages at different times (0, 12, 24, 48, and 72 h).

For TSP, an aliquot of 100 µL of the beverages was mixed with sodium carbonate and Folin-Ciocalteu reagent, and the absorbance at 765 nm was measured on a Perkin Elmer lambda 2 UV–VIS spectrophotometer, according to Cilla et al. [19]. Quantification was performed using an external standard calibration curve of gallic acid in the range of 0–500 mg/L. The results were expressed as gallic acid equivalent (GAE)/L.

TEAC assay is a spectrophotometric method that measures the reduction in the radical cation ABTS by antioxidant compounds [20]. The absorbance of 2 mL of ABTS+ working solution was considered the initial point of reaction (A0). Then, diluted samples (from 1/1 to 1/20 *v*/*v*) or Trolox standard (100 µL) were added immediately, and the absorbance was measured after 3 min (Af). All measurements were carried out at 30 °C in a thermostatized UV–VIS spectrophotometer (Perkin Elmer lambda 2 UV–VIS, Überlingen, Germany). The percentages of absorbance inhibition were obtained from the following equation:

1 − (Af/A0) × 100(1)

The results were compared to the Trolox standard curve to express them as µM Trolox equivalents/L.

The ORAC assay is a fluorimetric method that measures the capacity of antioxidant compounds to scavenge peroxyl radicals. The reaction was carried out in a Multilabel Plate Counter VICTOR^3^ 1420 with fluorescence filters for an excitation wavelength of 485 nm and an emission wavelength of 535 nm at 37 °C, according to Cilla et al. [19]. Samples were diluted 1/50 (*v*/*v*). The final reaction consisted of 80 μL of fluorescein, 40 μL of AAPH) and 80 μL of the diluted sample, Trolox standard or phosphate buffer (blank) and the fluorescence was recorded every 5 min over 70 min (until the fluorescence in the assay was less than 5% of the initial value). The results were calculated considering the differences in areas under the fluorescence decay curve (AUC) between the blank and the sample over time and were expressed as µM Trolox equivalents/L.

### 2.7. Analysis of Phytochemicals Profileby UPLC-ESI-QTOF-MS/MS

Tiger nut, carob and rice beverages were extracted three times with 2 mL of methanol (MeOH) and centrifuged at 14,000× *g* for 5 min. The supernatant (6 mL) and pellet were separated. The pellet was re-extracted with 200 μL of dimethyl sulfoxide (DMSO) and 2 mL of MeOH, vortexed for 30 min at 60 °C and centrifuged for 5 min. This step was repeated three times for a total volume of 6 mL. Finally, combined supernatants (12 mL) were evaporated overnight in a speed vacuum concentrator (Savant SPD121P, Thermo Scientific, Alcobendas, Spain), re-suspended in 100 µL of MeOH and filtered through a 0.45 µm PVDF filter before analysis using an Agilent 1290 Infinity UPLC system coupled to the 6550 Accurate-Mass Quadrupole TOF Mass Spectrometer (Agilent Technologies, Waldbronn, Germany) using an electrospray interface with Jet Stream technology.

The chromatography and mass spectrometry conditions were like those previously described [21] with some modifications. Briefly, separation was achieved on a reverse phase Poroshell 120 EC-C18 column (3 × 100 mm, 2.7 µm; Agilent) operating at 30 °C. The mobile phases were water–formic acid (99.9:0.1 *v*/*v*; phase A) and acetonitrile (ACN)–formic acid (99.9:0.1 *v*/*v*; phase B). Spectra were acquired in single MS mode with m/z range of 100–1100, negative polarity, and an acquisition rate of 1.5 spectra/s. Internal mass calibration by simultaneous acquisition of reference ions and mass drift compensation was used to obtain low mass errors. The most interesting compounds were selected for targeted MS/MS analysis, which provides high confidence in compound identification. MS/MS product ion spectra were collected at a m/z range of 50–800 using a retention time window of 1 min, a collision energy of 20 V and an acquisition rate of 4 spectra/s. Data were processed using the Mass Hunter Qualitative Analysis software (version B.10.00 Agilent Technologies). A target screening strategy was applied to all the samples for the qualitative screening of possible phytochemical metabolites. This strategy consists of searching for a list of target compounds after MS full acquisition. The screening was based on mass filtering at the exact mass of the compound investigated using narrow mass extraction windows (0.01 m/z). The identification was possible with the valuable information given in the QTOF-MS acquisition mode that provides possible molecular formulae for the compounds based on the accurate mass and isotopic pattern. Besides, targeted MS/MS experiments offered fragmentation information, providing more confidence in the compound identification process. When possible, a direct comparison with authentic standards was performed. Data on the identification of phytochemicals by UHPLC-QTOF are provided as (Supplementary Material Tables S1–S3 for tiger nut, carob and rice beverages, respectively).

### 2.8. Proximate Composition of the Beverages

All analytical measurements were performed according to the official method. The moisture content of the three beverages was determined at 95 °C in an oven (Memmert, Ule 500AO, Schwabach, Germany), using a temperature below 100 °C to avoid caramelization due to the significant amount of sugars [22]. The ash content was determined by incineration at 550 °C according to the official method [23] in a muffle furnace (Heraeus K1253, Hanau, Germany). Due to the high carbohydrate content, a ramp of 200 °C for 4 h was added before the incineration process to achieve proper carbonization of the carbohydrates. The temperature increased by 100 °C each hour until the incineration temperature remained for 8 h. Finally, the samples were cooled in a desiccator for 1 h and weighed. The total dietary fiber content was determined using a commercial K-TDFR-100A/K kit (Bray, Ireland) according to the official method [24]. The lipid content was determined using the modified Folch method [25]. For the determination of the fatty acid profile, an alkaline derivatization (KOH in methanol) was performed on the extracted fat, followed by hexane extraction. One µL of the extract was injected in a Clarus590 gas chromatograph (PerkinElmer, Shelton, CT, USA) equipped with a column SUPELCO (Burlington, PA, USA) SP^®^-2560; 100 m × 0.25 mm × 0.20 µm). The injector was set at 250 °C and the FID detector at 255 °C. The starting temperature of the oven was 180 °C for 5 min, followed by a rise to 210 °C for 5 min and finally 250 °C for 20 min. The carrier gas (N_2_) flow was set at 1.0 mL/min. Protein determination was carried out using the Kjeldahl method [26], applying the following conversion factors: 6.25 (for tiger nut beverages), 5.95 (for rice beverages), and 5.71 (for carob beverages) [27]. The carbohydrate content was determined by difference according to the formula:

Carbohydrates = 100 − [% (*w*/*w*) moisture + ash + total dietary fiber + lipids + proteins] (2)

All analytical measurements were performed in triplicate, except for the fiber assay, which was performed in duplicate according to the official method.

### 2.9. Sensory Attributes

A preliminary sensory test was carried out with 11 non-trained panelists. The panelists gave their consent to take part in the study and for us to use their information, and the appropriate protocols for protecting the rights and privacy of all participants were used. Samples of unfermented and fermented rice, carob and tiger nut beverages were named with random codes and offered to the panelists in an isolated room. The panelists had to rate the beverages according to the aroma descriptors “acid”, “sour”, “sweet”, “fruity”, “rancid”, “herbal”, and “floral” in values ranging from 0 (attribute not present) to a maximum of 3 (present and very intense).

### 2.10. In Vitro Static Gastrointestinal Digestion

The fermented tiger nut, carob and rice beverages underwent an in vitro gastrointestinal digestion procedure according to the INFOGEST 2.0 method [28] in order to evaluate the survival of the LAB and, thus, their probiotic potential. The simulated salivary fluid (SSF), simulated gastric fluid (SGF), and simulated intestinal fluid (SIF) were prepared using this method. Briefly, 5 g of each fermented beverage was mixed with 3.5 mL of SSF, 0.5 mL of α-amylase solution (to obtain a final concentration of 75 U/mL), 25 µL of 0.3 M calcium chloride and 975 µL of ultrapure water to obtain a final volume of 10 mL. The oral bolus was placed in a shaker bath for 2 min at 37 °C and 95 rpm. Upon completion of the oral phase, 7.5 mL of SGF, 0.98 mL of rabbit gastric extract (to obtain a final concentration of 60 U lipase/mL), 0.62 mL of pepsin solution (to obtain a final concentration of 2000 U/mL) and 5 µL of 0.3 M calcium chloride were added and mixed manually for one min. The pH of the mixture was adjusted to 3, and ultrapure water was added up to a volume of 20 mL. The gastric mixture was placed again in a shaker bath for 2 h under the same conditions. To simulate the intestinal conditions, 11 mL of SIF, 5 mL of pancreatin solution (to obtain a final concentration of 100 U/mL), 40 µL of 0.3 M calcium chloride, and 2.5 mL bovine bile solution (to obtain a final concentration of 10 mM) were added to gastric digesta. The final mixture was manually stirred for one min, adjusted to pH 7, and ultrapure water was added to a final volume of 40 mL. Finally, digesta was incubated in a shaking bath for 2 h at 37 °C and 95 rpm and subsequently centrifuged (90 min, 4 °C, 3100× *g*) to obtain the supernatant, corresponding to the bioaccessible fraction. Digestions were performed in duplicate.

### 2.11. Survival of LAB Consortia

The bioaccessible fractions from the digestion of the fermented beverages were analyzed to determine the presence of viable bacteria after in vitro digestion (INFOGEST 2.0), as described in Section 2.5.

### 2.12. Statistical Analysis

Results are generally expressed as the mean ± standard deviation from 3–5 replicates in at least two different experiments. For data with multiple groups, an analysis of variance (ANOVA) followed by post hoc tests, such as Tukey’s multiple comparison test or the Least Significant Difference (LSD) test, were carried out. For analyses that do not involve groups, a Student’s t-test was performed. In all instances, statistical significance was established if the null hypothesis was rejected at the *p* < 0.05 level.

## 3. Results and Discussion

### 3.1. Fermentation Process

**Figure 1:**
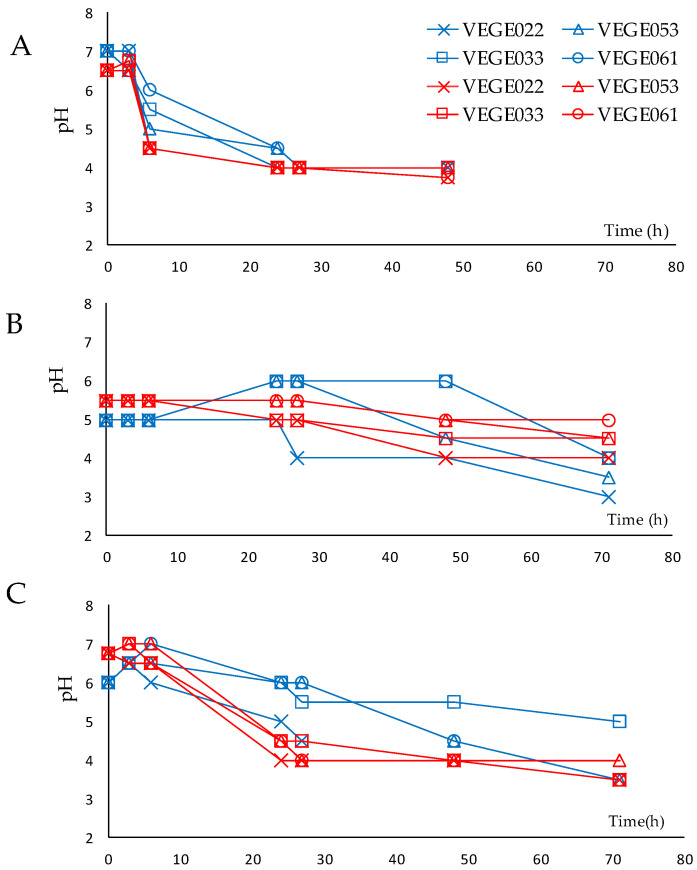
pH evolution throughout fermentation with different commercial starters of lactic acid bacteria at 30 °C (blue lines) or 37 °C (red lines) in tiger nut (**A**), carob (**B**), and rice (**C**) beverages. The standard deviation among samples was ±15%.

The unfermented beverages had an initial amount of soluble solids expressed in degrees Brix (°Brix) of 5.5 for the tiger nut beverage, 4.5 for the rice beverage and 3.5 for the carob beverage. In general, these beverages have a low percentage of soluble solids, as reported by other studies, and the nature of these can vary significantly depending on the source matrix [29]. For example, in the case of rice, despite containing high levels of starch, it also has maltose that can promote fermentation [11]. In the case of tiger nut, with regard to carbohydrates, there are twice as many sugars as starch due to the intrinsic activity of α-amylase [30], and this could favor a faster fermentation than rice. On the other hand, carob has high amounts of simple carbohydrates [14]. However, the percentage of soluble solids in this beverage was the lowest, probably because large amounts of solids cannot be extracted during the industrial brewing process. Fermentation kinetics (pH and °Brix) and microbial counts were analyzed at 30 °C and 37 °C for the time required for the beverages to reach a pH of 4–4.5. Figure 1 shows pH values during fermentation, while Table 2 presents the microbial counts. These data indicate that 37 °C yielded higher microbial counts and faster pH reduction.

After the initial fermentation periods at 30 °C (48–72 h), neither pH nor colony counts reached levels of 4–4.5. Viable counts in the beverages were quite discontinuous between starters and food matrix, probably due to the low initial Brix degrees. This suggests that the matrix may play an important role in LAB survival during fermentation. For this reason, it was decided to perform a new fermentation process from scratch, maintaining the temperature at 37 °C and adding three levels of glucose to try to improve fermentation kinetics and microbial counts. According to previous studies, it was determined that the addition of a carbon source (glucose) could help in the fermentation process [11,31]. On the other hand, there is no definitive consensus on the exact amount required to indicate that food is considered probiotic, but generally, food supplements vary from 1 × 10^6^ to 1 × 10^8^ CFU/g or mL [32]. This amount is necessary to assure the survival of probiotics to the gastric phase, although this survival also depends on the matrix, the type of microorganism and the interaction with other foods, among other factors [32].

**Figure 2:**
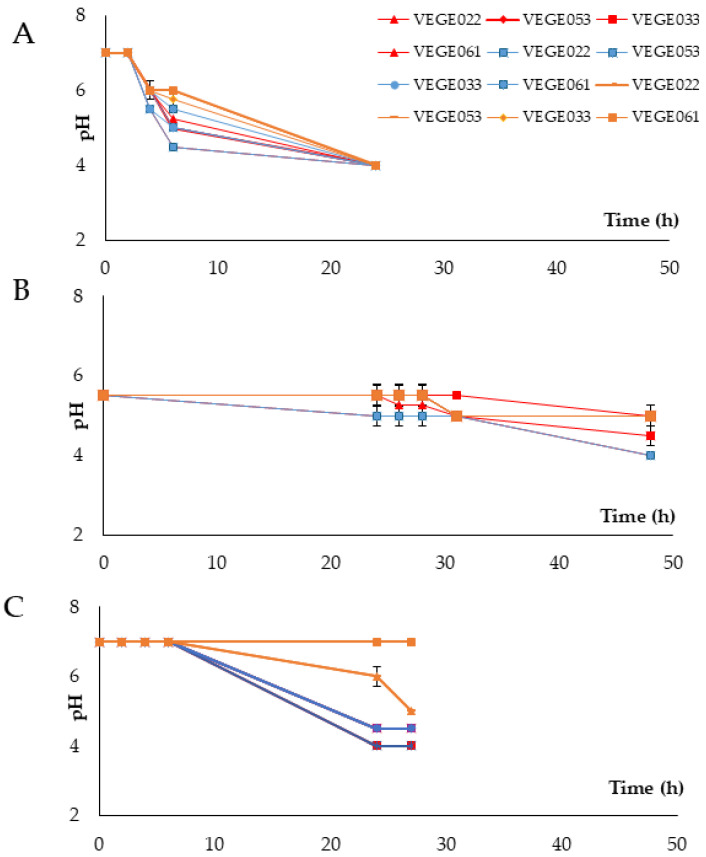
pH evolution throughout fermentation with different commercial starters of lactic acid bacteria at 37 °C in tiger nut (**A**), carob (**B**), and rice (**C**) beverages. The red lines indicate sugar level 1 (no glucose addition), the blue lines indicate sugar level 2 (7.5 g/100 mL of glucose for rice and tiger nut beverages and 15 g/100 mL for the carob beverage), and the brown lines indicate sugar level 3 (15 g/100 mL of glucose for the rice and tiger nut beverage and 30 g/100 mL for the carob beverage).

The differences in pH and microbial counts based on the addition of sugar are shown in Figure 2 and Table 2, respectively. Across all three beverages, with sugar level 2 (7.5 g of sugar/100 mL for rice and tiger nut beverages and 15 g/100 mL for carob beverage), the best microbial counts and the fastest pH drop were achieved. Sugar levels 1 and 3 resulted in higher pH values and lower microbial counts, being particularly notable in level 3, where high glucose concentrations likely caused significant inhibition of microbial growth due to osmotic stress. Therefore, the optimal fermentation conditions were set at 37 °C and level 2 of sugar addition.

### 3.2. Antioxidant Capacity Parameters

**Figure 3:**
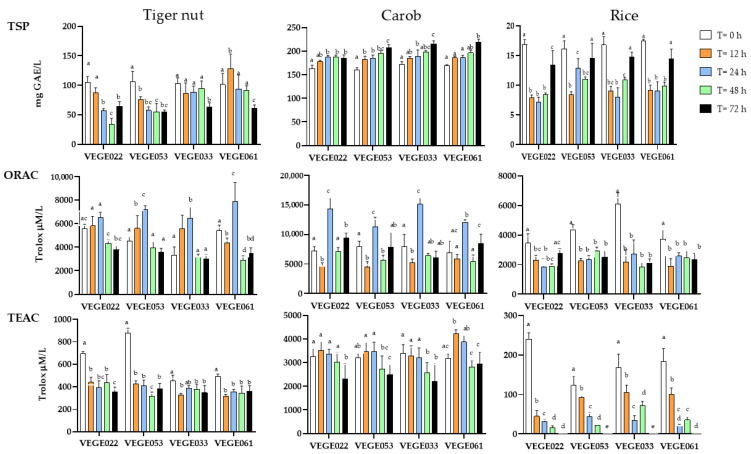
Changes in total antioxidant capacity (TSP, ORAC and TEAC assays) over time (0–72 h) in tiger nut, carob and rice fermented beverages with four different starters of LAB. Different letters (a–e) indicate statistically significant differences within the same LAB starter according to the ANOVA-Tukey test at a 95% significance level (*p* value < 0.05).

Once the temperature and sugar level adequate for efficient fermentation were verified, the antioxidant potential was determined by TSP, ORAC and TEAC methods on all the fermented beverages at different times (0, 12, 24, 48 and 72 h) to establish the best time-consortium combination with greater antioxidant potential. The results showed that carob beverage had the highest total antioxidant capacity in all tests, followed by tiger nut and, finally, rice beverages (Figure 3). The best antioxidant capacity values were obtained, in general, at 72 h for carob and 12–24 h for rice and tiger nut fermented beverages. Among the four starters used for fermentation, VEGE061 is generally the most promising in obtaining beverages with higher antioxidant content.

#### 3.2.1. Total Soluble Polyphenols

According to our results, TSP content differed depending on the food matrix, fermentation time, and the LAB starter used in fermentation. Regarding the type of matrix, TSP followed the order: carob > tiger nut > rice (159.8–218.9 > 34.1–127.9 > 7.2–17.5 mg GAE/L, respectively).

In the fermented tiger nut beverage, the best results (127.9 mg GAE/L) were obtained with the VEGE061 consortium after 12 h of fermentation, although, in general, TSP values were similar or lower after fermentation compared to the unfermented beverage. Our results for the unfermented tiger nut beverage (101.4–105.7 mg GAE/L) were higher than those reported in other studies in this type of matrix. For instance, Llorens et al. [33] found TSP values of 60.3 mg GAE/L for “horchata” beverages, which is an important source of antioxidants. Furthermore, Badejo et al. [16] found that in tiger nut beverages obtained from fresh tiger nut extract, the phenolic content was 21.7 mg/100 mL, and confirmed that germination and roasting significantly increased TSP, while the addition of *Hibiscus sabdariffa* and *Moringa oleifera* did not. Regarding fermentation, Satir [12] aimed to evaluate the physicochemical changes after fermentation with water kefir grains, which also contain, among other microorganisms, *L. acidophilus*, *S. thermophilus*, and *L. plantarum* in two varieties of tiger nut (yellow and brown) and their respective “horchata” beverages. They obtained GAE values close to 100 mg GAE/L, and although a slight increase in TSP values was detected, these changes were not statistically significant, in agreement with our results specifically for consortia VEGE053 and VEGE061, while we detected a significant decrease with consortia VEGE022 and VEGE033. In the aforementioned study, TSP was only determined at time 0 and at the end of fermentation. The authors did not specify the required fermentation time but assumed optimal fermentation when pH reached 4.6.

Regarding carob beverages, fermentation led to a generally significant increase in TSP among all LAB starters and fermentation times, from 159.8 to 218.9 mg GAE/L. The best results were obtained with consortia VEGE053, VEGE033, and VEGE061 after 72 h (206.9–218.9 mg GAE/L). These TSP values were the highest compared to the other two tested beverages, which was in accordance with the fact that carob appears to possess superior antioxidant capacity compared to many cereals, fruits, and vegetables [34]. The pulp, which is the main part used, not only has a high carbohydrate content but also contains phenolic compounds [35]. The lack of studies on carob beverages limits the discussion of these results. One study explored the physicochemical, antioxidant, and microbiological changes in a dairy beverage supplemented with carob (4%) and fermented with *Lactococcus lactis* C15 and *Lactobacillus brevis* B13 and B38 at 30 °C for 16 h. The results showed a significant increase in TSP from 1.12 to 5.48 mg GAE/g after fermentation, with this positive trend continuing during storage at 4 °C until reaching 6.2 mg GAE/g at 28 days [13]. Rodriguez et al. analyzed two traditional carob beverages (*Prosopis alba*): Aloja (traditionally fermented) and Añapa (unfermented). The beverages were prepared with white carob pods (4:96 pods–water *w*/*v*), and in the case of Aloja, fermented for 10 days at 25 °C in darkness using only the natural microbiota present in the pods, without specifying or characterizing the microorganisms involved. Although the results showed that Aloja presented significantly higher TSP content (150–180 mg GAE/L) compared to Añapa (21 mg GAE/L), it is difficult to establish direct comparisons with studies using specific starter cultures such as LAB. The increase in TSP was attributed to higher solubility in the ethanol produced during fermentation (5.2% at 3 days and 6.7% at 10 days), but the lack of microbial characterization limits the understanding of the specific mechanisms involved in this process [14].

Finally, in the case of rice beverages, TSP appears to decrease with fermentation in all samples, with the smallest decrease observed after 72 h of fermentation, where there is a tendency to recover initial values. Rice showed the lowest TSP values among the three analyzed beverages (7.2–17.5 mg GAE/L), probably due to the fact that white rice is not a significant source of phenolic compounds, especially compared to brown rice [36]. Additionally, it is important to consider that beverages represent a more diluted form of the whole food, which could further explain the low presence of total polyphenols. There is a notable lack of studies evaluating physicochemical changes in 100% plant-based, non-alcoholic beverages made exclusively from rice. In this context, Zou et al. [37] evaluated the physicochemical characteristics of rice beverages fermented for 24 h at 30 °C with three strains of *Pediococcus pentosaceus* (DH16, DH20 and DH24 in a 1:1:1 ratio) and supplemented with chestnut, which significantly improved antioxidant capacity. The fermented rice beverage batch without chestnut supplementation had an average TSP content of 90.14 mg GAE/L, while that including chestnut supplementation reached 131.17 mg GAE/L. Santos et al. [38] evaluated antioxidant changes during the production of amazake (a traditionally fermented beverage made with rice and *Aspergillus oryzae*) enriched with chestnut. Fermentation was carried out for 24 h at 50 °C. Using cooked chestnut and commercial rice amazake as controls, chestnut-enriched and non-enriched amazake samples showed statistically similar values of TSP (6.95, 6.56, 6.28, and 5.13 mg GAE/g, respectively). However, the authors suggested that these similar values could be due to limitations of the method used to quantify TSP, which would not detect the flavonols present in the chestnut samples.

#### 3.2.2. Oxygen Radical Absorbance Capacity Assay (ORAC)

In accordance with TSP results, the tiger nut beverages also ranked second in antioxidant capacity in the ORAC assay. Values ranged between a minimum of 2916.8 and a maximum of 7897.3 µM Trolox/L. The lowest values were reached after 48 and 72 h of fermentation, while maximum values were detected at 24 h of fermentation in all consortia, with a notable increase in the VEGE061 consortium. Maximum ORAC levels correspond to the optimal fermentation time as established in 3.1. Zhu et al. [39] found values of 1210 µM Trolox/L in unfermented tiger nut beverage, lower than those in our study. Unfortunately, no additional articles were found evaluating ORAC in tiger nut or its derivatives, with or without fermentation.

The carob beverages presented the highest ORAC levels compared to rice or tiger nut beverages, with values ranging from 4461.9 µM Trolox/L up to a maximum of 15,111.6 µM Trolox/L, found at 24 h of fermentation and for the VEGE033 consortium, followed by LAB starters VEGE022 and VEGE061. After reaching these maximum levels, the values decreased at 48 h, followed by an increase at 72 h, especially with the VEGE022 and VEGE061 consortia, a moment that coincides with the drop in pH at 72 h of fermentation. A study by Rodríguez-Solana et al. [40] evaluated the antioxidant capacity of various liquors obtained from carob through different extraction techniques (hydroalcoholic infusion, maceration, percolation, aqueous infusion, and distillation), showing values similar to our study (ranging from 2 to 6 mmol Trolox/L), with higher values in maceration and lower in distillation. The only study that evaluated ORAC in fermented carob-based beverages found values between 350 and 476 μmol Trolox/L during fermentation (24 h, 30 °C) and subsequent in vitro digestion in a dairy beverage fermented with *L. brevis* and supplemented with 4% carob powder [13]. Differences in probiotic strain type, fermentation conditions, and food matrix may explain these different values.

The rice beverages showed the lowest values in the ORAC assay, with values ranging between 1845.7 and 6103.5 µM Trolox/L. This is not surprising, as other studies have shown that among the cereals most commonly used in foods, rice generally has the lowest antioxidant capacity [41]. The fermentation process also caused a significant decrease in ORAC values versus the unfermented beverage, which remained constant from 12 to 72 h. Jung et al. [42] analyzed the antioxidant properties of 21 different varieties of fermented rice bran, whose results showed an increase in ORAC values after fermentation with the *Lentinula edodes* fungal mycelium. However, these findings differ from ours, considering the matrix difference (not a beverage) and the use of fungi instead of LAB for fermentation. Although there are studies exploring the fermentative viability of LAB in rice beverages, many of them do not evaluate their antioxidant capacity, or the analyzed beverages have high ethanol concentrations or are complex mixtures beyond simple rice as the sole ingredient. Additionally, some components derived from microbial fermentation of these traditional rice beverages, such as organic acids, enzymes, and other metabolites, could significantly affect their antioxidant capacity, as demonstrated by the high content of phenolic compounds and strong antioxidant capacity observed in beverages such as Haría, Xaj-pani or Sake [4,15].

#### 3.2.3. Trolox Equivalent Antioxidant Capacity (TEAC) Assay

In the tiger nut beverages, values ranged between 318.9 and 876.7 µM Trolox/L, showing a decrease after fermentation, and similar values were observed for all LAB starters at 12 h of fermentation. Studies directly assessing the antioxidant properties of tiger nut beverages are scarce, and even fewer, including fermentation, all of them with variable results. In one of these studies, Hernández-Olivas et al. [43] found values of 44.8 μmol Trolox/g and 24.4 μmol Trolox/g in tiger nut beverages without and with added sugars, respectively, while Llorens et al. [33] found 12.5 μmol TE/g in unfermented rice beverage. Additionally, Badejo et al. [16] found a 13% inhibition of ABTS+ radical in “horchata” made from fresh tiger nut extract, with a slight increase in inhibition after tuber germination. Regarding fermentation and contrary to our results, Satir [12] found a significant increase in TEAC values after fermentation, obtaining values of 4.61 and 5.98 mM Trolox/mL for yellow and brown variety beverages, respectively, and values of 7.55 and 7.96 mM Trolox/mL for their corresponding fermented beverages. This fact implies that the effect of fermentation on antioxidant compounds is LAB species-specific and also depends on fermentation conditions and the type of food matrix.

The carob beverages showed the highest TEAC values compared to rice and tiger nut beverages, ranging between 2057.7 and 4235.3 µM Trolox/L. The maximum value was found at 12 and 24 h of fermentation with the VEGE061 consortium. Furthermore, considering that the optimal fermentation time for carob beverages is 72 h, a slight decrease in the TEAC assay was also observed at this time. Rodríguez-Solana [40] found TEAC values higher than ours in carob infusions around 29,210 μmol Trolox/L, probably due to the high extraction efficiency and considering that our beverage contains only a small percentage of carob. Additionally, Rodríguez et al. [14] found that in Aloja, TEAC antioxidant capacity increased throughout 3 to 12 days of fermentation compared to Añapa, although there were no statistically significant differences between days 3 and 12. This fact suggests that fermentation, as well as the presence of alcohol, may induce higher antioxidant capacity, but only during the initial days of fermentation, after which this capacity is stabilized. Demarinis et al. [44] found a significant increase in the values measured by DPPH on fermentation with *L. plantarum* in carob flour. As mentioned previously, it is challenging to compare these results with other studies due to the scarcity of research on fermented carob-based beverages.

The highest TEAC values were found in the rice beverages for unfermented samples. After the fermentation process, there was a significant decrease in antioxidant capacity with all consortia, with values falling from 239.7 µM Trolox/L before fermentation until they became undetectable at 72 h of fermentation. The smallest decrease in antioxidant capacity was observed at 12 h of fermentation. To our knowledge, there are no studies on the impact of fermentation on the TEAC values of rice beverages. However, da Silva et al. [45] aimed to evaluate the physicochemical changes in a new rice beverage made from different grains (white, red, and black). The researchers demonstrated that the beverage had much lower values compared to whole grains but still exhibited activity against the ABTS+ radical, with values ranging from 3 to 10 µM Trolox/g for white and black rice, respectively, and intermediate values for red rice. These values are higher than ours for the unfermented beverage. 

### 3.3. Phytochemical Compounds

A metabolomic assay was conducted using Ultra-High-Performance Liquid Chromatography coupled with Quadrupole Time-of-Flight Mass Spectrometry (UHPLC-QTOF) to explore the complex mixture of compounds in detail and identify those contributing to antioxidant capacity, thus providing a more comprehensive understanding of their potential health benefits. The analyses were performed at 24 h fermentation for tiger nut and rice and 72 h for carob beverages, as these were the optimal fermentation times in terms of pH decrease and generally rendered the highest values of antioxidant capacity.

A total of 19 phytochemicals were detected in both fermented and unfermented tiger nut beverages, as shown in Table 3 (corresponding retention times are reported in Table S1).

Notably, there was a significant increase in homovanillic acid in the VEGE022 samples compared to the unfermented beverages. This increase may be attributed to the action of *Lactobacillus plantarum.* Shan et al. [46] demonstrated that fermentation of hemp seeds with *L. plantarum* significantly increased the production of homovanillic acid, which in turn had the highest inhibitory effect on inflammatory cytokines such as TNF-α, IL-6, IL-1β, and NO. Similar results were obtained by Zao et al. [47] with barley extract fermentation. Several studies have explored the metabolic profile of tiger nuts. Pelegrin et al. [48], using mass spectrometry with multiple reaction monitoring, analyzed dry tiger nut powder and identified seven compounds: 4-hydroxybenzaldehyde, p-coumaric acid, ferulic acid, sinapic acid, cinnamic acid, luteolin, and naringenin. The only compound common with our results was ferulic acid, which significantly increased with VEGE033 and mostly with VEGE061 LAB starter. Conversely, Saeed et al. [49], using UHPLC-ESI-QTOF MS on *C. esculentus* extracts, identified a total of 97 different compounds, including saccharides, amino acids, organic acids, fatty acids, phenolic compounds, and flavonoids. Citric acid, ferulic acid, and hydroxylinoleic acid were also found in our results. Hydroxy and dihydro derivatives of fatty acids can naturally occur in plants or be products of bacterial metabolism [50]. These compounds constitute a complex class of metabolites, some of which appear to have anti-inflammatory and anti-diabetic activity [51,52]. Instead, our study detected L-leucine acid, known to be a product of bacterial metabolism [53]. L-leucine acid appears to have interesting metabolic activity, such as degrading murine myotubes or affecting cell cycle arrest in Jurkat cells [54,55]. Our results also showed a significant decrease in kaempferol 3′-7′ diglucoside in fermented samples and a slight increase, especially in VEGE022, in ethyl vanillin. These compounds were also detected in by-products of the tiger nut industry [56]. In addition, these substances can present varied isomeric or conjugated forms depending on the variety used and have been associated with antioxidant, chemopreventive, and cardiovascular protective activities [57,58]. Taking together our results, the fluctuations observed during fermentation at 24 h (increase in homovanillic acid, ferulic acid and ethyl vanillin and decrease in kaempferol 3′-7′ diglucoside) could explain the general increase in ORAC values at 24 h and a decrease or maintenance of TSP and TEAC values (see Figure 3) since these antioxidants could be more effective against the peroxyl radicals of the ORAC method. On the other hand, a total of 47 different compounds were detected in the fermented and unfermented carob beverages (Table 4), the beverage type with the highest complexity and proportion of phytochemical compounds compared to rice and tiger nut beverages. This fact can justify their highest antioxidant capacity values observed.

Of these 47 compounds, 28 were phenolic compounds, confirming that this beverage contains a high phenolic content [59]. Santonocito et al. [60] aimed to evaluate the phytochemical profile of red and green carob seed extracts and identified 17 compounds by LC-MS. Among these, only gallic acid and quercetin were also found in our results. Gallic acid significantly decreased with fermentation in the VEGE053 and VEGE061 samples. Gallic acid is indeed one of the main phenolic compounds in carob [61], which, according to some reports, is one of the richest natural sources of this compound, second only to chestnut and clove [37]. Carob fruit contains a wide range of phenolic compounds, including phenolic acids, various flavonoids, and tannins. Our main phenolic acids were gallic acid and ellagic acid. Herein, ellagic acid significantly decreased after fermentation in contrast to other studies that reported that fermentation with *L. plantarum* strains of pomegranate peel infusion significantly increased its level [62]. The literature also reports the presence of other phenolic acids, such as caffeic, ferulic, chlorogenic, and cinnamic acids, but in lower quantities [63]; however, these were not detected in our study. As for flavonoids, the main compounds reported in the literature are quercetin, kaempferol, and myricetin [37]. In our study, we were able to detect and quantify all three. We also detected other flavonoids, such as quercitrin, luteolin, and isorhamnetin. The latter two were detected only in fermented samples, and several studies have shown that these flavonoids can increase significantly after fermentation [64,65]. In line with this, a total of 16 compounds were detected in the fermented samples that were not found in those unfermented, supporting the hypothesis that fermentation contributes to the release of various phytochemicals, increasing their detection even if not statistically significant when compared to unfermented samples. In fact, the increase after 72 h fermentation of phenolic compounds such as cynaroside A, gallic acid 4-O-(6-galloylglucoside), gallotannin (and isomer), delphinidin 3-O-3”,6-O-dimalonylglucoside, benzoic acid, kaempferide 7-glucoside, eriodictyol, luteolin, isorhamnetin and 3′-5′-dihydroxyflavanone may explain the increase in TSP and ORAC values (see Figure 3). Additionally, phytochemicals in their conjugated forms, such as quercetin 3-*O*-glucoside, quercetin 3-*O*-arabinoside, and apigenin 7-*O*-glucoside, were detected. These showed decreased concentrations in all fermented samples, and although the changes were not statistically significant, they could indicate partial degradation of these phytochemicals by LAB. Other researchers have also found a wide range of phenolic compounds in various carob-based syrups. For example, Zannini et al. [66] identified 76 different compounds, of which over 90% were hydroxybenzoic acids, with gallic acid being the main one, followed by ellagic acid. Both were detectable and quantifiable in our study. Other compounds were detected but not in all samples, as the researchers analyzed carob syrups from different commercial sources, indicating that variety and production methods can alter the phytochemical profile. Studies on the phytochemical profile of fermented carob-based products are scarce. Other researchers have found that certain compounds can be completely degraded during simulated in vitro digestion to undetectable levels, as reported by Ortega et al. [67], who observed complete degradation of certain compounds, such as ferulic acid in carob matrices, consistent with the fluctuations observed in our results for phenolic compounds. Rodriguez et al. [14] evaluated the phytochemical profile in alcoholic and non-alcoholic fermented beverages and found similar HPLC profiles with no significant differences between the two beverages. However, in our study, some compounds decreased with fermentation, such as gallic acid and isoquercetin (3-*O*-glucoside of quercetin). Similar results were obtained by Ait Chait [13], who evaluated changes in polyphenolic levels in fermented dairy beverages enriched with carob flour. The researchers found that the carob flour induced a significant change in antioxidant capacity, with 13 phenolic compounds responsible for this. They also observed that phenolic content decreased proportionally with the number of days of refrigeration, reaching the lowest values at 28 days. The same researchers evaluated the role of both digestion and fermentation on carob polyphenols, both in their soluble and bound forms. The authors found that free phenolics increased considerably, while conjugated and bound phenolics were partially degraded over time under digestion conditions [68]. These findings could explain the fluctuations in polyphenol content and antioxidant capacity observed in our carob beverages during different fermentation times.

Finally, in the case of rice beverages, a total of 15 compounds were detected (two of them phenolic compounds), which is shown in Table 5. This lowest level of phytochemicals among the three beverages confirms its lowest antioxidant capacity (see Figure 3).

The fermented samples showed a decrease in citric acid. This compound can be utilized by LAB to produce aromatic compounds such as diacetyl [69]. Besides, fermentation also led to a significant reduction in p-coumaric acid levels. Other studies have found that certain LAB, particularly *L. plantarum*, decreased the amount of p-coumaric acid in various fermented plant-based beverages [70,71]. However, not all phytochemicals decreased with fermentation in our study. We observed an increase in ethyl vanillin and sinapyl alcohol compared to the non-fermented samples, especially with the VEGE061 LAB starter. These findings are consistent with results from other studies through various transformations, such as the conversion of carbohydrates or dietary fiber [70,72]. In general, these fluctuations in the phenolic compounds may explain the decrease in TSP, ORAC, and TEAC found after fermentation with all LAB starters at 24 h (Figure 3), indicating that probably the degradation of these antioxidant compounds prevailed over the increase in some others on the final outcome in total antioxidant capacity. The last eight compounds identified were hydroxylated derivatives of fatty acids. In our fermented samples, there was a significant decrease in the levels of these compounds. Another study has shown that this can vary greatly depending on the strain used. Fiorino et al. [73] extensively evaluated the production of hydroxyl and epoxy acids in nut fermentation with LAB, both inter- and intra-species. Specifically, for our LAB starters, the researchers found that *S. thermophilus* significantly reduced the production of hydroxyl acids, while *L. paracasei* and *L. plantarum* induced different effects. *L. paracasei* decreased all hydroxyl acids but increased hydroxylinolenic acid, whereas *L. plantarum* reduced all hydroxyl acids but increased hydroxyl linolenic and hydroxyl linoleic acids, the latter of which was not found in our study. It is known that rice is a source of phenolic compounds, as reported by previous reviews [74]. Simulated in vitro digestion has been shown to significantly decrease the bioaccessibility of compounds such as p-coumaric acid in rice flour, although it turned out to be, among all cereal proteins, the one that suffers the least from the digestion process [75]. The observed variations in phytochemical profiles among different consortia and matrices reflect the complex interaction between microorganisms and substrates. The environment significantly influences microbial metabolism, and the interaction between microbial strains influences their adaptation to the environment, specifically the production of enzymes necessary to decompose the substrate [76]. The variability in results could be attributed to the heterogeneity of analytical methods used in different laboratories. Additionally, phytochemical variability is highly dependent on the specific strain or subspecies used for fermented products.

### 3.4. Proximate Chemical Composition

After selecting the appropriate fermentation parameters (temperature, sugar levels, and fermentation time), taking into account pH decrease, probiotic viability and antioxidant potential, proximate composition analysis was carried out on all the initial beverages and those fermented with all LAB starters. These analyses determine the nutritional composition of the beverages. Therefore, it is possible to identify differences depending on the microbial consortium. Table 6 shows the results of proximate composition for the fermented and non-fermented beverages for the three raw materials employed in this study.

The moisture content (%, *w*/*v*) of the beverages was 86.2–87.0% for tiger nut, 81.9–96.3% for carob, and 87.7–98.7% for rice beverages. In the case of carob and rice beverages, a significant decrease in moisture after fermentation with all LAB starters was observed, which was directly linked to an increase in carbohydrates and, consequently, in the caloric value. This pattern was particularly notable in rice beverages, where the moisture content decreased from 98.68% to around 88%, corresponding with an increase in caloric value from 3.17 to 45.99 kcal. In the tiger nut beverages, the ash content ranged between 0.045–0.051%, being lower in the case of carob (0.024–0.028%) and showing more variation in rice (0.016–0.035%) beverages. Regarding macronutrients, the fat content varied significantly among the analyzed beverages: tiger nut showed the highest values (1.2–1.8%), while carob presented the lowest (0.04–0.07%), and rice maintained intermediate levels (0.2–0.4%). The fiber values were generally very low in all the beverages, with no detectable values with VEGE061 consortium for carob and VEGE022 for rice, suggesting that the fermentation process might affect fiber content differently depending on the substrate. Eke-Eijofor et al. [77] found slightly lower protein and fat values in tiger nut beverages compared to our results but with slightly higher moisture and ash values. These small differences could be attributed to variations in the industrial process (e.g., higher grinding, lower filtration, etc.) or to the origin of the raw material. As for carob beverages, the comparison with the literature has proven to be a challenge, as there is a scarcity of available studies on 100% plant-based carob beverages. Recent reviews [4,78] compiled all available information on carob-based products; despite appearing in a wide variety of products, most have a dairy base or are made with a large number of ingredients based on homemade or traditional preparations. Elfazizi et al. [59] prepared carob beverages in the laboratory but only analyzed moisture and ash content. These authors obtained moisture values similar to ours and significantly higher ash content. This discrepancy might be explained by their use of a 3:1 water-to-pulp ratio, whereas ours was 9:1, highlighting the importance of standardizing preparation methods in future research. The carob drink also had the lowest protein content of all the beverages tested. Although no similar studies were found, the manufacturing process and the degree of dilution could partly explain these differences and may also have acted as a limiting source of nitrogen for a faster fermentation process, as it reached the appropriate pH within 72 h. Rodriguez et al. [14] provided information on sugars and proteins in Aloja and Añapa, which were similar to our findings. Finally, in the case of rice, our composition did not differ significantly from commercial samples, although da Silva et al. [45] found slightly higher values for protein (ranging from 1.14 ± 0.11% to 1.75 ± 0.00%) and ash (ranging from 0.12 ± 0.01% to 0.36 ± 0.03%) compared to ours, possibly due to differences in rice varieties or processing methods.

#### Fatty Acid Profile

**Figure 4:**
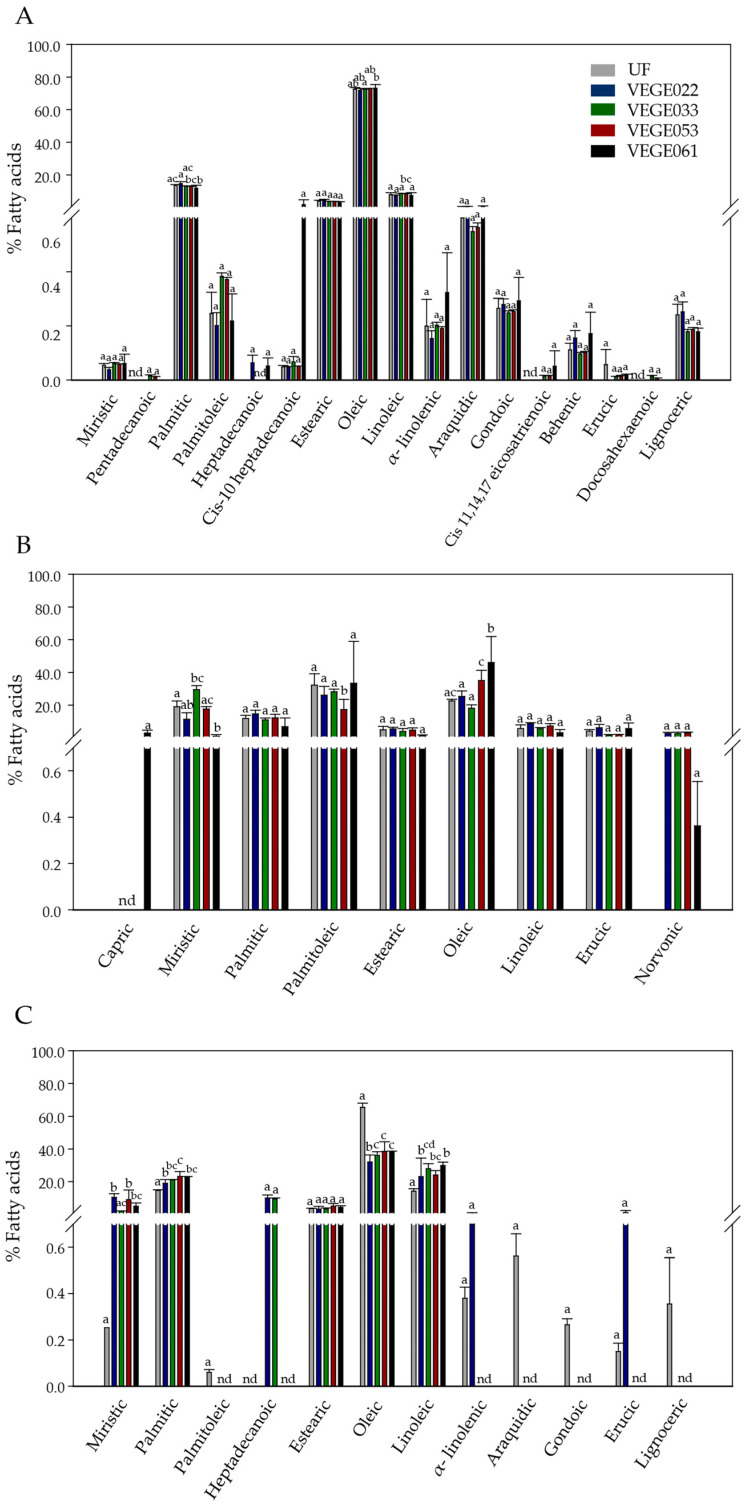
Fatty acid profiles of unfermented and fermented beverages with the four microbial consortia of LAB in tiger nut (**A**), carob (**B**), and rice (**C**) beverages. Different letters (a–d) indicate statistically significant differences according to the ANOVA–Tukey test at a 95% significance level (*p* value < 0.05). nd: not detected.

The complexity of fatty acids was greater in tiger nut beverages, followed by those made with rice and carob (Figure 4). Other researchers reported on the variety of fatty acid profiles in tiger nut beverages. Durman et al. [79] found 17 different types of fatty acids in tiger nut seeds similar to ours but with slight differences. In our study, neither arachidonic acid nor Cis-11-eicosenoic, Cis-11,14-eicosadienoic, Cis-8,11,14-eicosatrienoic, myristoleic or gamma linoleic fatty acids were found. In turn, in our study, pentadecanoic, cis-10 heptadecanoic, gondoic, behenic, erucic acids were detected. Cis 11,14,17 eicosatrienoic, behenic, erucic, and very small amounts of docosahexaenoic were found only in the fermented versions, thus determining a more mono, polyunsaturated profile compared to these authors. Other authors who evaluated the fatty acid profile after extraction of the lipid phase with supercritical CO_2_ in tiger nut beverage “horchata” found a variety of 13 fatty acids in similar proportion to ours, with the only exception that we did not find C18:1n-7 acid [80]. There is a lack of evidence of the role of fermentation on changes in the fatty acid profile in tiger nut derivatives.

Oleic acid was the main fatty acid in tiger nut and rice beverages, while palmitoleic acid predominated in carob beverages. Fermentation produced changes in the lipid profile depending on the bacterial consortium used. The fatty acid profile in tiger nut beverages revealed that the percentage of monounsaturated fatty acids (MUFA), mainly oleic acid, increased with the VEGE061 consortium. Small amounts of cis-11,14,17 eicosatrienoic acid and docosahexaenoic acid were also detected after fermentation with the VEGE022 and VEGE033 consortia. Carob beverages showed an increase in saturated fatty acids (SFA) with the VEGE033 consortium, while there was a significant decrease, reaching 12% with the VEGE061 consortium. Polyunsaturated fatty acids (PUFA) increased significantly with the VEGE061 consortium compared to non-fermented beverages, with the monounsaturated fatty acids reaching 85%, although MUFA remained the main fatty acids in all samples as reported by other authors in non-fermented carob samples [81]. In rice beverages, a drastic increase in SFA, particularly in the production of myristic and palmitic acids and polyunsaturated fatty acids (PUFA), was observed, with a notable decrease in monounsaturated fatty acids (MUFA). However, with VEGE061, these changes were less pronounced, maintaining the general profile of the non-fermented beverages, which is MUFA > SFA > PUFA. There is a lack of studies evaluating specific changes in fatty acid profile in fermented rice beverages with LAB. Some authors evaluate rice beverages without fermentation and found great variety in the proportion of fatty acids depending on the rice variety used and the processing method, such as germination, with oleic acid concentrations that could vary between 5 and 85%. The reported values for linoleic and linolenic acids fall within our ranges [82].

### 3.5. Sensory Attributes

**Figure 5:**
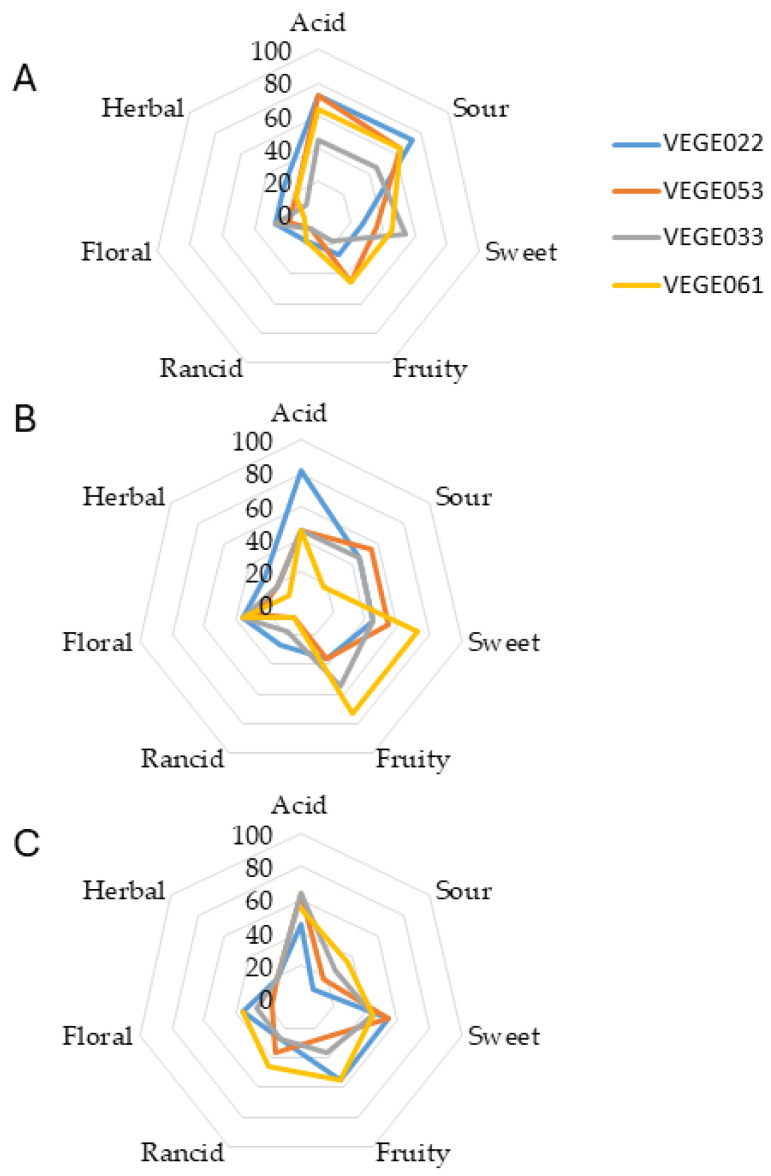
Sensory analysis of tiger nut (**A**), carob (**B**), and rice (**C**) beverages fermented at 37 °C with level 2 glucose addition (7.5 g/100 mL of glucose for rice and tiger nut beverages and 15 g/100 mL for the carob beverages).

After the optimal fermentation time had elapsed, the beverages underwent a preliminary analysis to assess their aroma with a panel of untrained tasters. The results obtained using a qualitative descriptive analysis are shown in Figure 5.

In the case of tiger nut beverages, fermentation allowed the production of ‘lactic/buttery’ and ‘fruity/floral’ aromas. This fact aligns with the metabolic activity of LAB, which generates diacetyl and esters responsible for these sensory characteristics [83]. Fermentation with *L. plantarum* in a tiger nut yogurt with 20% date palm achieved the highest acceptability scores [84]. However, consortium VEGE061 exhibits more rancid notes, likely due to lipid oxidation or undesirable by-products common in fat-rich substrates. For carob beverages, fermentation significantly alters the sensory profile, particularly in ‘cheese/sweat’ and ‘undergrowth’ aromas, possibly due to the presence of isobutyric acid, as reported by other studies [85,86]. VEGE061 shows a more balanced aroma, indicating better strain performance. Additional research has shown that fermentation with *L. plantarum* in carob flour intensifies the cocoa aroma and decreases the astringent and earthy notes, which is consistent with our results for the VEGE061 consortium, which contains several *Lactobacillus* strains and showed a more balanced aroma profile [44]. Considering rice beverages, their carbohydrate-rich composition offers a favorable matrix, with VEGE033 and VEGE061 showing a positive aroma balance, highlighting their potential for delivering probiotics with appealing sensory qualities [87].

### 3.6. Viable Counts After Digestion

**Figure 6:**
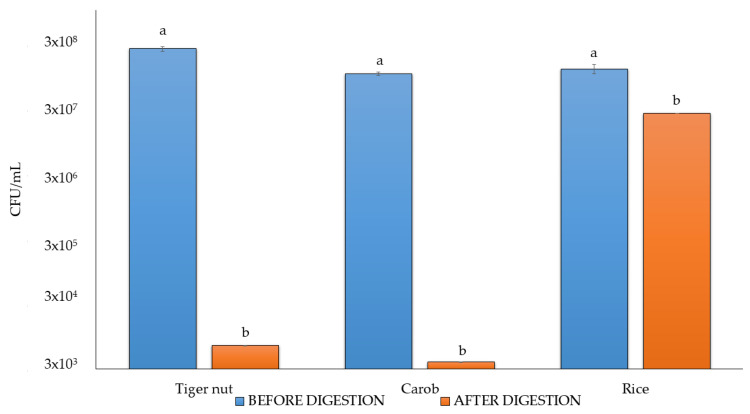
Viable counts before and after digestion for tiger nut, carob and rice beverages fermented with the VEGE061 consortium. Different letters (a, b) in the same sample indicate statistically significant differences (*p* value < 0.05) according to Student’s *t*-test.

Taking into consideration the optimal fermentation time, the higher antioxidant values, and more pleasant sensory attributes, beverages fermented with the VEGE061 consortium were selected to ascertain the viability of probiotics after digestion. Figure 6 shows the viable count before and after the in vitro simulated gastrointestinal digestion.

Microbial viability decreased after digestion by more than four logarithmic units for carob and tiger nut beverages, whereas just one logarithmic unit reduction was found in rice beverages. Similar findings were reported in other studies evaluating the in vitro viability of commercial lyophilized LAB during gastrointestinal digestion, where reductions of 1–3 log units in probiotic viability are typically expected [32,88]. Therefore, the observed reduction of over four logarithmic units for carob and tiger nut is slightly higher than typically reported but within a plausible range given the matrix and processing conditions. Based on these findings, rice could be considered a more suitable matrix for delivering probiotics, as the probiotics demonstrate better survival during the digestive process. However, the use of adjuvants or some form of encapsulation could be considered to achieve high probiotic viability across all three matrices. Despite this, Treven et al. demonstrated that commercial encapsulation methods using regular capsules, tablets and powder formulations were not effective against gastric conditions, and the administration form significantly impacted probiotic survival, with porridge matrix showing better results (91.8%) compared to juice (79.0%) [89]. Despite the significant decrease in viability for carob and tiger nut, these beverages could still offer other health benefits, such as acting as substrates for the fermentation of endogenous strains (prebiotic-like) or exhibiting postbiotic activity [90]. Nonetheless, in vivo trials would be needed to confirm these potential benefits.

## 4. Conclusions

This study has provided several significant insights into the fermentation of tiger nuts, carob, and rice beverages using different LAB starters. Firstly, fermentation at 37 °C was found to be more effective than at 30 °C, significantly enhancing LAB activity and resulting in a pH of around 4 with a high viable count (>10^8^ CFU/mL). Additionally, optimal sugar concentrations of 7.5% for tiger nut and rice and 15% for carob beverages were crucial for effective fermentation, as they support LAB growth and metabolism. The impact of fermentation on antioxidant capacity and phytochemicals was dependent on the food matrix and LAB starter. Hence, carob beverages exhibited the highest antioxidant capacity, attributed to their rich polyphenol content and the complexity of phytochemicals. In addition, the antioxidant capacity, in general, peaked at 24 h for rice and tiger nut and at 72 h for carob beverages, indicating that fermentation time plays a critical role in enhancing (or maintaining) antioxidant levels. The VEGE061 provided, in general, the best outcome. Regarding nutritional composition, tiger nuts showed the highest fat content (1.2–1.8%). The fatty acid profile analysis revealed that the VEGE061 consortium increased MUFA, particularly oleic acid, in tiger nut beverages while promoting higher SFA in rice beverages. The sensory evaluation demonstrated distinctive profiles for each beverage: tiger nuts developed desirable lactic/buttery and fruity/floral notes, especially with VEGE033 and VEGE061; carob, fermented with VEGE061, showed a balanced aroma profile; and rice achieved pleasant sensory qualities, particularly with VEGE033 and VEGE061 consortia. Following simulated in vitro gastrointestinal digestion, a significant decline in probiotic counts was observed in tiger nut and carob beverages, while higher viability was exhibited in rice beverages. This suggests that, although the LAB consortium was effective in fermentation, the survival of bacteria could depend on the matrix and formulation of the product. These findings underscore the importance of optimizing fermentation conditions and highlight the complex phytochemical transformations that occur during fermentation. Despite the comprehensive approach of this study, several limitations should be acknowledged. The number of bacterial consortia tested was limited to four commercial starters, which may not represent the full spectrum of potential LAB combinations. Additionally, the significant reduction in probiotic viability after in vitro digestion, particularly in tiger nut and carob beverages, suggests that protective strategies such as microencapsulation techniques should be explored. The relatively short fermentation period (up to 72 h) and the absence of storage stability assessments also represent areas for improvement. Furthermore, laboratory-scale production might not fully reflect the challenges of industrial-scale manufacturing. These limitations provide clear directions for future research, which should focus on the following: (1) exploring a wider range of LAB strains and customized consortia; (2) developing improved probiotic protection methods specific to each plant matrix; (3) investigating extended fermentation periods and storage conditions; (4) scaling up production processes; and (5) evaluating the bioaccessibility of antioxidant phytochemical compounds and the bioactivity of these fermented and unfermented beverages in preclinical models to unravel their potential beneficial health effects. Our research group is currently addressing several of these aspects to build upon the foundation established in this study.

## Figures and Tables

**Table 1:** Composition of the commercial starters.

Danisco^®^ VEGE022	Danisco^®^ VEGE033	Danisco^®^ VEGE053	Danisco^®^ VEGE061
*Streptococcus* *thermophilus* *Lactobacillus* *delbrueckii* subsp. *bulgaricus* *Lactobacillus* *acidophilus* NCFM^®^ *Bifidobacterium* *animalis* subsp. *lactis* HN019 ^®^ *Lactobacillus plantarum*	*Streptococcus* *thermophilus* *Lactobacillus* *delbrueckii* subsp. *bulgaricus*	*Streptococcus* *thermophilus* *Lactobacillus* *delbrueckii* subsp. *bulgaricus* *Lactobacillus* *acidophilus* NCFM^®^ *Bifidobacterium* *animalis* subsp. *lactis* HN019 ^®^	*Streptococcus* *thermophilus* *Lactobacillus* *delbrueckii* subsp. *bulgaricus* *Lactobacillus* *acidophilus* NCFM^®^ *Bifidobacterium* *animalis* subsp. *lactis* HN019 ^®^ *Lactobacillus* *paracasei*

**Table 2:** Colony counts (CFU/mL) after fermentations at different temperatures (30 and 37 °C) and different levels of glucose addition for tiger nut, carob and rice beverages.

Sample	Starter	30 °C	37 °C	Level 1 *	Level 2 **	Level 3 ***
Tiger nut	VEGE022	1.38 × 10^8^ ± 1.17 × 10^8^	3.00 × 10^8^ ± 3.15 × 10^7^	3.70 × 10^8^ ± 1.00 × 10^7^	4.25 × 10^8^ ± 1.50 × 10^7^	4.50 × 10^8^ ± 3.00 × 10^7^
	VEGE033	2.05 × 10^7^ ± 1.20 × 10^6^	1.02 × 10^8^ ± 3.00 × 10^6^	4.45 × 10^8^ ± 3.50 × 10^7^	4.50 × 10^8^ ± 0	2.50 × 10^8^ ± 1.50 × 10^6^
	VEGE053	1.77 × 10^5^ ± 1.25 × 10^4^	3.00 × 10^8^ ± 0	2.06 × 10^8^ ± 2.55 × 10^7^	2.15 × 10^8^ ± 1.65 × 10^7^	2.35 × 10^8^ ± 3.85 × 10^7^
	VEGE061	3.59 × 10^7^ ± 3.51 × 10^7^	1.04 × 10^8^ ± 5.50 × 10^6^	3.95 × 10^8^ ± 1.65 × 10^8^	3.15 × 10^8^ ± 1.50 × 10^7^	6.30 × 10^8^ ± 1.10 × 10^8^
	VEGE022	1.26 × 10^6^ ± 7.05 × 10^5^	7.80 × 10^7^ ± 1.70 × 10^7^	8.35 × 10^7^ ± 8.50 × 10^6^	1.10 × 10^8^ ± 5.00 × 10^5^	9.35 × 10^7^ ± 4.50 × 10^6^
Carob	VEGE033	1.43 × 10^7^ ± 5.35 × 10^6^	1.08 × 10^7^ ± 2.00 × 10^5^	2.57 × 10^7^ ± 3.00 × 10^5^	8.00 × 10^7^ ± 1.00 × 10^7^	2.45 × 10^7^ ± 3.50 × 10^6^
	VEGE053	6.40 × 10^7^ ± 3.10 × 10^7^	5.60 × 10^5^ ± 2.80 × 10^5^	2.55 × 10^7^ ± 8.00 × 10^5^	3.70 × 10^7^ ± 1.00 × 10^6^	2.25 × 10^7^ ± 5.50 × 10^6^
	VEGE061	6.80 × 10^7^ ± 2.40 × 10^7^	4.92 × 10^7^ ± 2.88 × 10^7^	1.45 × 10^7^ ± 3.50 × 10^6^	4.30 × 10^7^ ± 1.60 × 10^7^	1.85 × 10^7^ ± 5.00 × 10^5^
	VEGE022	7.50 × 10^7^ ± 4.00 × 10^6^	7.00 × 10^7^ ± 2.00 × 10^6^	1.73 × 10^8^ ± 1.85 × 10^7^	1.45 × 10^8^ ± 3.50 × 10^7^	1.42 × 10^8^ ± 1.40 × 10^7^
Rice	VEGE033	4.09 × 10^7^ ± 3.41 × 10^7^	5.06 × 10^6^ ± 4.54 × 10^6^	4.70 × 10^6^ ± 9.00 × 10^5^	2.75 × 10^7^ ± 6.50 × 10^6^	3.60 × 10^6^ ± 4.00 × 10^5^
	VEGE053	4.50 × 10^7^ ± 1.40 × 10^7^	4.40 × 10^7^ ± 2.00 × 10^7^	5.85 × 10^6^ ± 2.05 × 10^6^	7.00 × 10^6^ ± 2.00 × 10^6^	1.15 × 10^6^ ± 5.00 × 10^4^
	VEGE061	1.47 × 10^8^ ± 1.15 × 10^8^	6.55 × 10^7^ ± 4.65 × 10^7^	9.75 × 10^6^ ± 5.00 × 10^4^	1.70 × 10^8^ ± 8.00 × 10^7^	n.d.

All tests at different sugar levels were conducted at 37 °C. Level 1 *: no glucose added. Level 2 **: 7.5 g/100 mL of glucose for rice and tiger nut beverages and 15 g/100 mL for carob beverages. Level 3 ***: 15 g/100 mL of glucose for rice and tiger nut beverages and 30 g/100 mL for carob beverages. n.d.: Not determined.

**Table 3:** Peak integration (×10^6^) area values from the EICs of the phytochemical compounds identified by UHPLC-QTOF in non-fermented and fermented tiger nut beverages employing different LAB consortia (VEGE022, VEGE033, VEGE053 and VEGE061) for 24 h.

No.	Metabolites	Non-Fermented	VEGE022	VEGE033	VEGE053	VEGE061
1	Citric acid	101.39 ± 4.00 ^a^	99.24 ± 2.52 ^a^	91.88 ± 7.32 ^a^	92.06 ± 10.13 ^a^	111.63 ± 19.11 ^a^
2	Homovanillic acid *	1.59 ± 0.97 ^a^	4.00 ± 0.36 ^b^	1.51 ± 0.49 ^a^	1.78 ± 1.17 ^a^	1.91 ± 0.70 ^a^
3	L-leucic acid	4.37 ± 0.20 ^a^	8.34 ± 0.29 ^b^	7.86 ± 1.92 ^b^	2.03 ± 0.69 ^a^	9.48 ± 1.77 ^b^
4	S-leucic acid	37.16 ± 1.60 ^a^	10.56 ± 0.20 ^b^	4.14 ± 1.59 ^c^	3.33 ± 1.57 ^c^	4.38 ± 1.10 ^c^
5	Kaempferol 3′,7-diglucoside *	1.46 ± 0.08 ^a^ **(3.30 ± 0.25 µM)**	1.18 ± 0.02 ^bc^ **(2.47 ± 0.07 µM)**	1.32 ± 0.12 ^ab^ **(2.88 ± 0.36 µM)**	1.02 ± 0.03 ^c^ **(1.97 ± 0.10 µM)**	1.06 ± 0.13 ^c^ **(2.09 ± 0.41 µM)**
6	4-vinylphenol *	2.81 ± 0.14 ^ab^	40.20 ± 0.04 ^b^	1.70 ± 0.47 ^ac^	2.38 ± 1.07 ^ac^	1.22 ± 0.23 ^c^
7	Ethyl vanillin *	19.20 ± 0.34 ^ab^	30.35 ± 0.22 ^b^	12.13 ± 4.43 ^a^	17.4 ± 8.55 ^a^	8.42 ± 2.02 ^a^
8	Ferulic acid *	n.d. ^a^	0.34 ± 0.01 ^a^	2.88 ± 2.07 ^bc^	0.59 ± 0.14 ^ac^	3.62 ± 0.43 ^c^
9	Sinapoyl alcohol	0.91 ± 0.06 ^a^	1.21 ± 0.01 ^a^	1.11 ± 0.17 ^a^	0.89 ± 0.25 ^a^	1.0 ± 0.36 ^a^
10	Dehydrodivanillin *	n.d. ^a^	0.89 ± 0.01 ^b^	0.92 ± 0.01 ^b^	0.58 ± 0.01 ^c^	0.60 ± 0.04 ^c^
11	Trihydroxy octadecenoic acid isomer a	28.89 ± 0.46 ^a^	28.35 ± 1.59 ^a^	30.27 ± 5.20 ^a^	17.70 ± 2.81 ^b^	13.01 ± 0.60 ^b^
12	Trihydroxy octadecenoic acid isomer b	22.69 ± 1.17 ^a^	20.62 ± 0.86 ^a^	22.69 ± 1.32 ^a^	12.20 ± 3.32 ^b^	6.20 ± 0.66 ^c^
13	Dihydroxyoleic acid isomer a	45.88 ± 0.88 ^a^	26.49 ± 1.61 ^b^	32.42 ± 5.75 ^b^	25.83 ± 1.54 ^b^	3.86 ± 3.60 ^c^
14	Dihydroxyoleic acid isomer b	43.76 ± 090 ^a^	26.96 ± 3.15 ^b^	27.09 ± 7.98 ^b^	19.90 ± 2.18 ^b^	3.02 ± 2.69 ^c^
15	Dihydroxystearic acid isomer a	18.43 ± 0.04 ^ab^	28.86 ± 17.43 ^b^	32.14 ± 0.12 ^b^	15.27 ± 1.60 ^ac^	1.95 ± 17.11 ^a^
16	Dihydroxystearic acid isomer b	71.66 ± 1.55 ^a^	84.16 ± 2.12 ^a^	81.49 ± 8.91 ^a^	71.82 ± 3.28 ^a^	14.28 ± 16.45 ^b^
17	Hydroxylinoleic acid isomer a	46.87 ± 0.46 ^a^	37.24 ± 5.45 ^ab^	27.20 ± 3.53 ^b^	40.02 ± 1.49 ^ab^	7.04 ± 9.47 ^c^
18	Hydroxyoleic acid isomer a	36.46 ± 0.80 ^a^	10.43 ± 0.99 ^b^	10.20 ± 3.28 ^bc^	15.75 ± 5.14 ^b^	2.30 ± 3.26 ^c^
19	Hydroxyoleic acid isomer b	3.91 ± 0.14 ^a^	1.46 ± 0.18 ^b^	2.92 ± 0.56 ^a^	3.24 ± 0.45 ^a^	0.36 ± 0.50 ^c^

* Phenolic compounds. EICs: Extracted Ion Chromatograms Metabolites. Metabolites highlighted in bold were quantified with external standards. n.d.: not detected. Different letters (a–c) in the same row indicate statistically significant differences (*p* value < 0.05) according to one-way ANOVA followed by LSD post-hoc test. Bold value indicates that phenolic-derived metabolites were quantified with an external standard (µM).

**Table 4:** Peak integration (×10^6^) area values from the EICs of the phytochemical compounds identified by UHPLC-QTOF in carob beverages non-fermented and fermented with different consortiums of LAB (VEGE022, VEGE033, VEGE053 and VEGE061) for 72 h.

No	Metabolites	Non-Fermented	VEGE022	VEGE033	VEGE053	VEGE061
1	2-Dehydro-D-xylonate	19.27 × 10^6^ ± 0.35 ^a^	36.15 ± 3.98 ^b^	11.76 ± 12.50 ^a^	22.35 ± 1.13 ^ab^	21.93 × 10^6^ ± 0.96 ^ab^
2	Citric acid	19.27 × 10^6^ ± 1.00 ^ab^	12.25 ± 2.63 ^b^	37.38 ± 35.25 ^abc^	54.17 ± 7.24 ^ac^	62.97 × 10^6^ ± 0.70 ^c^
3	2-Deoxy-D-Ribose	43.56 × 10^6^ ± 1.00 ^a^	44.62 ± 2.82 ^a^	32.08 ±35.85 ^a^	45.55 ± 11.68 ^a^	52.22 × 10^6^ ± 1.44 ^a^
4	(R)-2-Methylmalate	20.69 × 10^6^ ± 0.77 ^a^	33.79 ± 2.86 ^a^	27.05 ± 12.88 ^a^	26.97 ± 10.41 ^a^	32.12 × 10^6^ ± 5.02 ^a^
5	2-Galloylglucose *	19.91 × 10^6^ ± 0.64 ^a^	1.82 ± 0.10 ^b^	1.80 ± 0.15 ^b^	1.83 ± 0.12 ^b^	10.09 × 10^6^ ± 11.92 ^ab^
6	Succinic acid *	16.13 × 10^6^ ± 0.15 ^a^	15.68 ± 1.09 ^a^	13.17 ± 6.36 ^a^	14.19 ± 2.74 ^a^	15.36 × 10^6^ ± 0.80 ^a^
7	2-Methylcitrate	13.00 × 10^6^ ± 0.03 ^a^	18.46 ± 1.61 ^a^	11.79 ± 15.23 ^a^	12.16 ± 10.83 ^a^	18.83 × 10^6^ ± 1.29 ^a^
8	Gallic acid *	98.84 × 10^6^ ± 1.97 ^a^	53.80 ± 61.87 ^ab^	98.48 ± 1.45 ^a^	12.42 ± 0.17 ^b^	12.57 × 10^6^ ± 0.64 ^b^
9	2-O-Galloylsucrose *	70.68 × 10^6^ ± 0.78 ^a^	105.35 ± 2.67 ^a^	57.77 ± 72.28 ^a^	87.87 ± 26.28 ^a^	107.79 × 10^6^ ± 0.47 ^a^
10	Phloroglucinol *	n.d.^a^	67.89 ± 4.31 ^b^	n.d. ^a^	n.d. ^a^	n.d. ^a^
11	Ethylmalonic acid	n.d. ^a^	19.79 ± 2.41 ^b^	12.10 ± 11.75 ^ab^	16.30 ± 5.94 ^b^	18.93 × 10^6^ ± 1.01 ^b^
12	b-D-Xylopyranosyl-(1-4)-a-L-rhamnopyranosyl-(1-2)-L-arabinose	21.28 × 10^6^ ± 1.64 ^a^	13.78 ± 0.34 ^a^	8.29 ± 9.94 ^a^	11.08 ± 5.58 ^a^	15.05 × 10^6^ ± 0.98 ^a^
13	a-L-Fucopyranosyl-(1-2)-b-D-galactopyranosyl-(1-2)-D-xylose	28.67 × 10^6^ ± 2.14 ^a^	49.37 ± 0.55 ^a^	25.20 ± 32.56 ^a^	55.94 ± 9.01 ^a^	63.31 × 10^6^ ± 2.93 ^a^
14	Cynaroside A *	n.d. ^a^	34.46 ± 0.51 ^b^	16.16 ± 22.57 ^ab^	24.35 ± 13.82 ^ab^	32.33 × 10^6^ ± 1.00 ^b^
15	3′-Methoxyfukiic acid	n.d. ^a^	17.47 ± 0.49 ^b^	11.95 ± 7.17 ^b^	16.01 ± 0.05 ^b^	15.35 × 10^6^ ± 0.16 ^b^
16	Gallic acid 4-O-(6-galloylglucoside) *	n.d. ^a^	33.81 ± 0.34 ^b^	18.62 ± 21.92 ^ab^	25.14 ± 12.04 ^ab^	23.62 × 10^6^ ± 0.68 ^ab^
17	3-propylmalic acid *	38.58 × 10^6^ ± 0.64 ^a^	48.32 ± 0.96 ^a^	34.11 ± 20.41 ^a^	30.26 ± 20.07 ^a^	40.31 × 10^6^ ± 1.96 ^a^
18	3-O-Methylgallate *	13.49 × 10^6^ ± 0.75 ^a^	6.32 ± 0.11 ^a^	24.95 ± 18.09 ^a^	8.99 ± 6.81 ^a^	11.70 × 10^6^ ± 0.75 ^a^
19	Eriocitrin *	22.60 × 10^6^ ± 1.06 ^a^	14.30 ± 0.27 ^ab^	7.89 ± 10.99 ^b^	10.90 ± 3.51 ^ab^	14.75 × 10^6^ ± 3.01 ^ab^
20	Gallotannin *	n.d. ^a^	49.03 ± 1.10 ^b^	28.75 ± 36.05 ^ab^	45.65 ± 13.48 ^b^	50.31 × 10^6^ ± 4.46 ^b^
21	Gallotannin (isomer) *	n.d. ^a^	47.24 ± 0.47 ^b^	28.97 ± 34.57 ^ab^	39.88 ± 18.85 ^ab^	50.24 × 10^6^ ± 4.29 ^b^
22	Delphinidin 3-O-3″,6″-O-dimalonylglucoside *	n.d. ^a^	11.62 ± 0.08 ^bc^	8.84 ± 3.38 ^b^	10.44 ± 0.82 ^b^	10.29 × 10^6^ ± 0.34 ^b^
23	Ellagic acid+ *	126.29 × 10^6^ ± 15.64 ^a^ **(126.05 ± 15.65 µM)**	71.10 ± 0.19 ^ab^ **(70.86 ± 0.19 µM)**	40.72 ± 44.40 ^b^ **(40.48 ± 44.40 µM)**	65.48 ± 10.63 ^b^ **(65.24 ± 10.63 µM)**	72.20 ± 0.90 ^ab^ **(71.96 ± 0.90 µM)**
24	Myricitrin+ *	25.83 × 10^6^ ± 0.99 ^a^ **(76.88 ± 2.98 µM)**	27.00 ± 0.64 ^a^ **(80.38 ± 1.92 µM)**	13.20 ± 18.27 ^a^ **(38.99 ± 54.81 µM)**	23.79 ± 4.68 ^a^ **(70.75 ± 14.05 µM)**	28.90 ±2.84 ^a^ **(86.09 ± 8.51 µM)**
25	Quercetin 3-O-glucoside+ *	15.38 × 10^6^ ± 1.62 ^a^ **(45.53 ± 4.87 µM)**	10.30 ± 0.66 ^ab^ **(30.29 ± 1.98 µM)**	5.01 ± 6.91 ^b^ **(14.42 ± 20.74 µM)**	9.79 ± 0.04 ^ab^ **(28.74 ± 0.13 µM)**	9.44 ± 0.59 ^ab^ **(27.70 ± 1.77 µM)**
26	Benzoic acid *	n.d. ^a^	19.86 ± 0.12 ^a^	10.58 ± 14.22 ^a^	11.11 ± 9.65 ^a^	16.85 ± 2.37 ^a^
27	Quercetin 3-arabinoside+ *	27.09 × 10^6^ ± 0.67 ^a^ **(80.64 ± 2.01 µM)**	18.18 ± 0.23 ^a^ **(53.93 ± 0.68 µM)**	11.52 ± 14.23 ^a^ **(33.93 ± 42.70 µM)**	15.81 ± 7.13 ^a^ **(46.82 ± 21.40 µM)**	22.08 ± 0.73 ^a^ **(65.63 ± 2.19 µM)**
28	Quercitrin+ *	150.54 × 10^6^ ± 4.97 ^a^ **(451.00 ± 14.92 µM)**	108.17 ± 0.76 ^a^ **(323.89 ± 2.28 µM)**	58.02 ± 79.22 ^a^ **(173.45 ± 237.66 µM)**	97.13 ± 22.39 ^a^ **(290.77 ± 67.17 µM)**	110.72 ± 5.87 ^a^ **(331.54 ± 17.61 µM)**
29	Isochinomin	14.13 × 10^6^ ± 0.86 ^a^	11.27 ± 0.40 ^a^	7.20 ± 10.03 ^a^	7.43 ± 5.60 ^a^	9.84 ± 0.22 ^a^
30	Kaempferide 7-glucoside *	n.d. ^a^	10.83 ± 0.56 ^b^	5.86 ± 7.94 ^ab^	10.91 ± 5.60 ^b^	11.80 ± 1.08 ^b^
31	Phloretin 2′-O-glucuronide+ *	67.98 × 10^6^ ± 4.01 ^a^ **(203.32 ± 12.03 µM)**	55.37 ± 1.21 ^a^ **(165.50 ± 3.64 µM)**	29.63 ± 41.40 ^a^ **(88.28 ± 124.22 µM)**	53.95 ± 7.61 ^a^ **(161.24 ± 22.84 µM)**	55.12 ± 0.20 ^a^ **(164.76 ± 0.61 µM)**
32	Apigenin 7-O-glucoside+ *	19.16 × 10^6^ ± 0.73 × 10^6 a^ (56.87 ± 2.19 μM)	12.26 ± 1.41 ^ab^ **(36.15 ± 4.24 µM)**	7.04 ± 9.31 ^b^ **(20.50 ± 27.93 µM)**	11.47 ± 2.31 ^ab^ **(33.79 ± 6.95 µM)**	11.85 ± 0.07 ^ab^ **(34.92 ± 0.21 µM)**
33	6-Hydroxykaempferol+ *	22.91 × 10^6^ ± 1.96 ^a^ **(68.12 ± 5.89 µM)**	26.75 ± 0.38 ^a^ **(79.64 ± 1.14 µM)**	14.70 ± 20.54 ^a^ **(43.49 ± 61.62 µM)**	23.43 ± 1.84 ^a^ **(69.66 ± 5.52 µM)**	20.51 ± 0.73 ^a^ **(60.90 ± 2.19 µM)**
34	Eriodictyol *	n.d. ^a^	212.65 × 10^6^ ± 0.56 ^a^	11.28 ± 15.74 ^a^	15.47 ± 9.00 ^a^	21.11 ± 5.84 ^a^
35	Luteolin *	n.d. ^a^	99.00 ± 1.40 ^b^	50.26^6^ ± 69.82 ^ab^	92.15 ± 9.25 ^b^	96.96 ± 0.70 ^b^
36	Quercetin+ *	28.86 × 10^6^ ± 1.19 ^a^ **(85.97 ± 3.57 µM)**	36.35 ± 6.77 ^a^ **(108.44 ± 20.31 µM)**	15.64 ± 21.70 ^a^ **(46.31 ± 65.10 µM)**	23.61 ± 11.89 ^a^ **(70.20 ± 35.68 µM)**	28.27 ± 17.01 ^a^ **(84.18 ± 51.05 µM)**
37	Isorhamnetin+ *	n.d. ^a^	21.44 ± 0.65 ^b^ **(63.71 ± 1.95 µM)**	9.18 ± 12.80 ^ab^ **(26.93 ± 39.39 µM)**	15.64 ± 3.06 ^b^ **(46.31 ± 9.19 µM)**	19.87 ± 3.26 ^b^ **(58.99 ± 9.77 µM)**
38	9S,12S,13S-trihydroxy-10E-octadecenoic acid	59.05 × 10^6^ ± 1.31 ^a^	154.98 ± 2.15 ^a^	55.37 ± 77.59 ^a^	74.88 ± 86.75 ^a^	132.46 ± 3.12 ^a^
39	3′,5′-Dihydroxyflavanone *	n.d. ^a^	17.14 ±0.16 ^b^	11.61 ± 7.60 ^b^	16.40 ± 0.64 ^b^	16.56 ± 0.46 ^b^
40	Octadecanedioic acid	44.63 × 10^6^ ± 0.85 ^a^	25.19 ± 1.17 ^ab^	11.64 ± 16.08 ^b^	15.81 ± 12.17 ^b^	24.06 ± 0.70 ^ab^
41	9,10-DiHOME	57.42 × 10^6^ ± 1.82 ^a^	36.72 ± 0.85 ^ab^	16.78 ± 21.98 ^b^	23.09 ± 11.12 ^b^	22.96 ± 7.78 ^b^
42	L-Menthyl acetoacetate	n.d. ^a^	11.73 ± 1.24 ^a^	6.84 ± 9.43 ^a^	10.67 ± 4.77 ^a^	10.91 ± 0.17 ^a^
43	9,10-dihydroxy stearic acid	n.d. ^a^	44.72 ± 6.50 ^b^	26.97 ± 33.54 ^ab^	42.40 ± 10.13 ^b^	44.06 ± 6.36 ^b^
44	Laserpitin	n.d. ^a^	15.29 ± 0.52 ^a^	12.32 ± 16.56 ^a^	18.01 ± 6.28 ^a^	14.11 ± 2.33 ^a^
45	alpha, alpha’-Trehalose 6-palmitate	22.41 × 10^6^ ± 0.80 ^a^	6.23 ± 0.26 ^b^	5.31 ± 7.34 ^b^	9.42 ± 1.84 ^b^	10.02 ± 1.41 ^b^
46	12R-hydroxy-9Z-octadecenoic acid	16.94 × 10^6^ ± 0.69 ^a^	56.62 ± 0.71 ^a^	32.22 ± 42.29 ^a^	41.43 ± 30.28 ^a^	59.85 ± 2.83 ^a^
47	16-hydroxy hexadecanoic acid	32.18 × 10^6^ ± 2.91 ^ab^	3.03 ± 1.44 ^c^	6.73 ± 9.07 ^c^	13.10 ± 12.60 ^ac^	42.62 ± 12.02 ^b^

* Phenolic compounds. EICs: Extracted Ion Chromatograms Metabolites. Metabolites highlighted in bold were quantified with external standards. n.d.: not detected. Different letters (a–c) in the same row indicate statistically significant differences (*p* value < 0.05) according to one-way ANOVA followed by LSD post-hoc test. Bold value indicates that phenolic-derived metabolites were quantified with an external standard (µM).

**Table 5:** Peak integration (×10^6^) area values from the EICs of the phytochemical compounds identified by UHPLC-QTOF in rice beverages non-fermented or fermented with different consortiums of lactic acid bacteria (VEGE022, VEGE033, VEGE053 and VEGE061) for 24 h.

No.	Metabolites	Non-Fermented	VEGE022	VEGE033	VEGE053	VEGE061
1	Citric acid	7.36 ± 1.21 ^a^	2.29 ± 0.06 ^b^	2.01 ± 2.35 ^b^	0.93 ± 0.75 ^b^	2.84 ± 1.07 ^b^
2	L-leucic acid	0.30 ± 0.13 ^a^	0.33 ± 0.05 ^a^	1.34 ± 0.86 ^a^	1.19 ± 1.34 ^a^	2.14 ± 0.63 ^a^
3	S-leucic acid	0.32 ± 0.09 ^a^	3.29 ± 0.01 ^ab^	24.24 ± 8.47 ^bc^	18.24 ± 18.69 ^abc^	31.67 ±1.49 ^c^
4	p-coumaric acid *	1.91 ± 0.12 ^a^	0.20 ± 0.01 ^b^	0.87 ± 0.07 ^c^	0.86 ± 0.50 ^c^	0.86 ± 0.13 ^c^
5	Ethyl vanillin *	n.d. ^a^	14.38 ± 0.62 ^c^	5.09 ± 2.58 ^ab^	6.43 ± 6.12 ^ab^	8.17 ± 1.10 ^bc^
6	Sinapoyl alcohol	4.78 ± 0.37 ^ab^	2.80 ± 0.11 ^a^	6.42 ± 0.39 ^b^	4.22 ± 2.97 ^ab^	6.73 ± 0.454 ^b^
7	Trihydroxy octadecenoic acid isomer a	79.88 ± 94.54 ^a^	112.14 ± 8.77 ^a^	120.86 ± 1.10 ^a^	104.55 ± 38.82 ^a^	142.30 ± 3.81 ^a^
8	Trihydroxy octadecenoic acid isomer b	108.79 ± 92.68 ^a^	51.52 ± 6.75 ^b^	72.97 ± 3.38 ^b^	53.91 ± 24.22 ^b^	77.29 ± 5.28 ^ab^
9	Dihydroxyoleic acid isomer a	26.19 ± 2.76 ^a^	55.09 ± 2.22 ^a^	31.16± 4.17 ^a^	36.50± 3.08 ^a^	23.29 ± 29.70 ^a^
10	Dihydroxyoleic acid isomer b	57.21 ± 4.37 ^a^	56.98 ± 4.79 ^a^	36.85 ± 1.19 ^b^	51.56 ± 2.43 ^a^	61.67 ± 10.41 ^a^
11	Dihydroxystearic acid isomer a	6.5 ± 0.64 ^a^	17.44 ± 3.36 ^a^	9.27 ± 1.41 ^a^	14.24 ± 8.96 ^a^	9.37 ± 0.35 ^a^
12	Dihydroxystearic acid isomer b	9.83 ± 1.15 ^a^	12.92 ± 0.60 ^b^	1.94 ± 0.30 ^c^	2.30 ± 0.88 ^c^	1.68 ± 0.28 ^c^
13	Hydroxylinoleic acid isomer a	89.01 ± 14.77 ^a^	28.38 ± 6.32 ^b^	1.63 ± 0.50 ^c^	2.11 ± 0.64 ^c^	0.78 ± 0.88 ^c^
14	Hydroxyoleic acid isomer a	18.13 ± 0.24 ^a^	3.62 ± 0.15 ^b^	3.98 ± 0.05 ^b^	4.47 ± 0.50 ^b^	4.84 ± 0.57 ^b^
15	Hydroxyoleic acid isomer b	14.49 ± 3.10 ^a^	4.65 ± 0.13 ^b^	5.99 ± 0.72 ^b^	4.88 ± 0.96 ^b^	6.40 ±0.20 ^b^

* Phenolic compounds. EICs: Extracted Ion Chromatograms Metabolites. n.d.: not detected. Different letters (a–c) in the same row indicate statistically significant differences (*p* value < 0.05) according to one-way ANOVA followed by LSD post-hoc test.

**Table 6:** Proximate composition expressed in g/100 g in the non-fermented (NF) and fermented with different LAB consortium tiger nut, carob and rice beverages. Saturated fat is calculated as % of total fatty acids.

Sample	Starter	Energetic Value (kcal)	Total Fat	Saturated Fat	Carbohydrates	Protein	Total Fiber	Moisture	Ash
Tiger nut	NF	56.126 ± 0.332 ^a^	1.197 ± 0.045 ^a^	18.66 ± 0.45 ^a^	10.311 ± 0.218 ^a^	0.863 ± 0.0008 ^a^	0.031 ± 0.0063 ^a^	86.52 ± 0.0012 ^ab^	0.048 ± 0.0003 ^a^
	VEGE022	55.746 ± 1.648 ^a^	1.242 ± 0.112 ^a^	20.41 ± 0.10 ^a^	9.978 ± 0.227 ^a^	0.980 ± 0.100 ^a^	0.042 ± 0.0121 ^a^	86.66 ± 0.0033 ^ab^	0.047 ± 0.0004 ^a^
	VEGE033	55.006 ± 0.938 ^a^	1.320 ± 0.485 ^a^	17.96 ± 0.06 ^a^	9.787 ± 0.926 ^a^	0.840 ± 0.07 ^a^	0.260 ± 0.0082 ^a^	86.96 ±0.0036 ^a^	0.051 ± 0.0002 ^a^
	VEGE053	58.509 ± 0.884 ^a^	1.461 ± 0.014 ^a^	17.72 ± 0.21 ^a^	10.423 ± 0.225 ^a^	0.770 ± 0.07 ^a^	0.046 ± 0.0265 ^a^	86.17 ± 0.0026 ^b^	0.049 ± 0.0002 ^a^
	VEGE061	56.083 ^a^ ± 4.695 ^a^	1.831 ± 0.160 ^a^	16.70 ± 0.53 ^a^	10.207 ± 1.062 ^a^	0.770 ± 0.07 ^a^	0.071 + 0.0629 ^a^	86.61 ± 0.0020 ^ab^	0.045 ± 0.0002 ^a^
Carob	NF	10.210 ± 0.423 ^a^	0.064 ± 0.010 ^a^	35.60 ± 3.31 ^ac^	2.302 ± 0.121 ^a^	0.09 ± 0.00	0.231 ± 0.1898 ^a^	96.30 ± 0.0011 ^a^	0.028 ± 0.0002 ^a^
	VEGE022	61.703 ± 0.636 ^b^	0.071 ± 0.012 ^a^	39.98 ± 3.26 ^c^	15.129 ± 0.181 ^b^	0.12 ± 0.00 ^bc^	0.357 ± 0.2186 ^a^	83.45 ± 0.0016 ^b^	0.026 ± 0.000 ^a^
	VEGE033	63.342 ± 3.373 ^c^	0.060 ± 0.014 ^a^	44.18 ± 1.32 ^a^	17.083 ± 0.813 ^c^	0.102 ± 0.00 ^a^	0.017 ± 0.0125 ^a^	81.88 ± 0.008 ^c^	0.024 ± 0.0007 ^a^
	VEGE053	59.482 ± 0.153 ^b^	0.052 ± 0.004 ^a^	37.96 ± 2.06 ^ac^	14.671 ± 0.058 ^b^	0.072 ± 0.0073 ^a^	0.701 ± 0.1349 ^a^	83.63 ± 0.004 ^b^	0.027 ± 0.0002 ^a^
	VEGE061	58.016 ± 0.378 ^b^	0.044 ± 0.007 ^a^	13.71 ± 8.12 ^b^	14.283 ± 0.100 ^b^	0.102 ± 0.0128 ^ac^	n.d.^a^	84.68 ± 0.009 ^d^	0.025 ± 0.0002 ^a^
Rice	NF	3.170 ± 0.584 ^a^	0.186 ± 0.040 ^a^	19.52 ± 1.00 ^a^	n.d.^a^	0.256 ± 0.0466 ^ab^	0.01 ± 0.005 ^ab^	98.68 ^a^ ± 0.003 ^a^	0.016 ± 0.0007 ^a^
	VEGE022	34.572 ± 4.254 ^b^	0.257 ± 0.053 ^ab^	42.72 ± 11.8 ^b^	7.824 ± 1.163 ^c^	0.244 ± 0.0139 ^ab^	n.d. ^a^	90.92 ^b^ ± 0.001 ^b^	0.023 ± 0.07 ^b^
	VEGE033	41.859 ± 5.656 ^bc^	0.281 ± 0.099 ^ab^	36.07 ± 0.85 ^b^	9.567 ± 1.209 ^bc^	0.220 ± 0.0115 ^a^	0.02 ± 0.033 ^ab^	88.94 ^bc^ ± 0.001 ^a^	0.035 ± 0.0002 ^c^
	VEGE053	45.991 ± 0.459 ^c^	0.214 ± 0.000 ^ab^	37.33 ± 4.27 ^b^	10.777 ± 0.189 ^c^	0.298 ± 0.0154 ^b^	0.05 ± 0.0352 ^b^	87.68 ^c^ ± 0.004 ^c^	0.032 ± 0.0001 ^c^
	VEGE061	44.172 ± 0.937 ^c^	0.386 ± 0.0922 ^b^	32.03 ± 2.36 ^b^	9.735 ± 0.086 ^bc^	0.240 ± 0.0066 ^b^	0.03 ± 0.005 ^ab^	88.86 ± 0.002 ^bc^	0.035 ± 0.0007 ^c^

Different letters (a–c) in the same column and in the same sample indicate statistically significant differences (*p* value < 0.05) according to one-way ANOVA followed by LSD post-hoc test. n.d: Not detected.

## Data Availability

The data that support the findings of this study are available on reasonable request from the corresponding author.

## Supplementary Materials

The following supporting information can be downloaded at https://www.mdpi.com/article/10.3390/foods14091447/s1, Tables S1–S3. Identification of phytochemicals by UHPLC-QTOF for tiger nut, carob and rice beverages, respectively.

## References

[1] Donat M.G., Lowry A.L., Alexander L.V., O’Gorman P.A., Maher N. (2016). More Extreme Precipitation in the World’s Dry and Wet Regions. Nat. Clim. Change.

[2] Mirzabaev A., Bezner Kerr R., Hasegawa T., Pradhan P., Wreford A., Cristina Tirado von der Pahlen M., Gurney-Smith H. (2023). Severe Climate Change Risks to Food Security and Nutrition. Clim. Risk Manag..

[3] Turkmen N., Akal C., Özer B. (2019). Probiotic Dairy-Based Beverages: A Review. J. Funct. Foods.

[4] Vitali M., Gandía M., Garcia-Llatas G., Tamayo-Ramos J.A., Cilla A., Gamero A. (2023). Exploring the Potential of Rice, Tiger Nut and Carob for the Development of Fermented Beverages in Spain: A Comprehensive Review on the Production Methodologies Worldwide. Beverages.

[5] Mukherjee A., Breselge S., Dimidi E., Marco M.L., Cotter P.D. (2024). Fermented Foods and Gastrointestinal Health: Underlying Mechanisms. Nat. Rev. Gastroenterol. Hepatol..

[6] Koutsoumanis K., Allende A., Alvarez-Ordóñez A., Bolton D., Bover-Cid S., Chemaly M., De Cesare A., Hilbert F., Lindqvist R., EFSA Panel on Biological Hazards (BIOHAZ) (2024). Update of the List of Qualified Presumption of Safety (QPS) Recommended Microbiological Agents Intentionally Added to Food or Feed as Notified to EFSA 19: Suitability of Taxonomic Units Notified to EFSA until September 2023. EFSA J..

[7] Grau-Fuentes E., Rodrigo D., Garzón R., Rosell C.M. (2023). Understanding the Marketed Plant-Based Beverages: From Ingredients Technological Function to Their Nutritional Value. J. Funct. Foods.

[8] Badejo A.A., Damilare A., Ojuade T.D. (2014). Processing Effects on the Antioxidant Activities of Beverage Blends Developed from *Cyperus esculentus*, Hibiscus Sabdariffa, and Moringa Oleifera Extracts. Prev. Nutr. Food Sci..

[9] Roselló-Soto E., Barba F.J., Putnik P., Bursać Kovačević D., Lorenzo J.M., Cantavella-Ferrero Y. (2018). Enhancing Bioactive Antioxidants’ Extraction from “Horchata de Chufa” By-Products. Foods.

[10] THE 17 GOALS|Sustainable Development. https://sdgs.un.org/goals.

[11] Gan R.-Y., Shah N.P., Wang M.-F., Lui W.-Y., Corke H. (2016). Fermentation Alters Antioxidant Capacity and Polyphenol Distribution in Selected Edible Legumes. Int. J. Food Sci. Technol..

[12] Yang F., Chen C., Ni D., Yang Y., Tian J., Li Y., Chen S., Ye X., Wang L. (2023). Effects of Fermentation on Bioactivity and the Composition of Polyphenols Contained in Polyphenol-Rich Foods: A Review. Foods.

[13] Cichońska P., Ziębicka A., Ziarno M. (2022). Properties of Rice-Based Beverages Fermented with Lactic Acid Bacteria and Propionibacterium. Molecules.

[14] Satir G. (2022). The Effects of Fermentation with Water Kefir Grains on Two Varieties of Tigernut (*Cyperus esculentus* L). Milk. LWT.

[15] Chait Y.A., Gunenc A.G., Bendali F.B., Hosseinian F. (2021). Functional Fermented Carob Milk: Probiotic Variability and Polyphenolic Profile. J. Food Bioact..

[16] Rodríguez I.F., Cattaneo F., Zech X.V., Svavh E., Pérez M.J., Zampini I.C., Isla M.I. (2020). Aloja and Añapa, Two Traditional Beverages Obtained from Prosopis Alba Pods: Nutritional and Functional Characterization. Food Biosci..

[17] Fuloria S., Mehta J., Talukdar M.P., Sekar M., Gan S.H., Subramaniyan V., Rani N.N.I.M., Begum M.Y., Chidambaram K., Nordin R. (2022). Synbiotic Effects of Fermented Rice on Human Health and Wellness: A Natural Beverage That Boosts Immunity. Front. Microbiol..

[18] Prior R.L., Wu X., Schaich K. (2005). Standardized Methods for the Determination of Antioxidant Capacity and Phenolics in Foods and Dietary Supplements. J. Agric. Food Chem..

[19] Cilla A., Perales S., Lagarda M.J., Barberá R., Clemente G., Farré R. (2011). Influence of Storage and in Vitro Gastrointestinal Digestion on Total Antioxidant Capacity of Fruit Beverages. J. Food Compos. Anal..

[20] Re R., Pellegrini N., Proteggente A., Pannala A., Yang M., Rice-Evans C. (1999). Antioxidant Activity Applying an Improved ABTS Radical Cation Decolorization Assay. Free Radic. Biol. Med..

[21] Ávila-Gálvez M.Á., García-Villalba R., Martínez-Díaz F., Ocaña-Castillo B., Monedero-Saiz T., Torrecillas-Sánchez A., Abellán B., González-Sarrías A., Espín J.C. (2019). Metabolic Profiling of Dietary Polyphenols and Methylxanthines in Normal and Malignant Mammary Tissues from Breast Cancer Patients. Mol. Nutr. Food Res..

[22] Cortés C., Esteve M.J., Frıgola A., Torregrosa F. (2005). Quality Characteristics of Horchata (a Spanish Vegetable Beverage) Treated with Pulsed Electric Fields during Shelf-Life. Food Chem..

[23] Thiex N., Novotny L., Crawford A. (2012). Determination of Ash in Animal Feed: AOAC Official Method 942.05 Revisited. J. AOAC Int..

[24] Tada S., Innami S. (2007). A Simplified Modification of the AOAC Official Method for Determination of Total Dietary Fiber Using Newly Developed Enzymes. J. AOAC Int..

[25] Boselli E., Velazco V., Fiorenza Caboni M., Lercker G. (2001). Pressurized Liquid Extraction of Lipids for the Determination of Oxysterols in Egg-Containing Food. J. Chromatogr. A.

[26] Kjeldahl J. (1883). Neue Methode zur Bestimmung des Stickstoffs in organischen Körpern. Z. Für Anal. Chem..

[27] Merrill A.L., Watt B.K. (1955). Energy Value of Foods: Basis and Derivation.

[28] Brodkorb A., Egger L., Alminger M., Alvito P., Assunção R., Ballance S., Bohn T., Bourlieu-Lacanal C., Boutrou R., Carrière F. (2019). INFOGEST Static in Vitro Simulation of Gastrointestinal Food Digestion. Nat. Protoc..

[29] Beaulieu J.C., Reed S.S., Obando-Ulloa J.M., McClung A.M. (2020). Green Processing Protocol for Germinating and Wet Milling Brown Rice for Beverage Formulations: Sprouting, Milling and Gelatinization Effects. Food Sci. Nutr..

[30] Yu Y., Lu X., Zhang T., Zhao C., Guan S., Pu Y., Gao F. (2022). Tiger Nut (*Cyperus esculentus* L.): Nutrition, Processing, Function and Applications. Foods.

[31] Deziderio M.A., de Souza H.F., Kamimura E.S., Petrus R.R. (2023). Plant-Based Fermented Beverages: Development and Characterization. Foods.

[32] Naissinger da Silva M., Tagliapietra B.L., Flores V.d.A., Pereira dos Santos Richards N.S. (2021). In Vitro Test to Evaluate Survival in the Gastrointestinal Tract of Commercial Probiotics. Curr. Res. Food Sci..

[33] Llorens P., Flavia Chiacchio M., Tagliamonte S., Juan-García A., Pallarés N., Carlos Moltó J., Vitaglione P., Juan C. (2024). Potential Bioaccessibility and Bioavailability of Polyphenols and Functional Properties of Tiger Nut Beverage and Its By-Product during in Vitro Digestion. Food Funct..

[34] Goulas V., Stylos E., Chatziathanasiadou M.V., Mavromoustakos T., Tzakos A.G. (2016). Functional Components of Carob Fruit: Linking the Chemical and Biological Space. Int. J. Mol. Sci..

[35] Tounsi L., Kechaou N. (2021). Influence of pectinase and cellulase extracts on carob juice yield and quality. Life Sci. J..

[36] Ma Y., Zhang S., Rong L., Wu Z., Sun W. (2022). Polyphenol Composition and Antioxidant Activity of Japonica Rice Cultivars and Intake Status. Foods.

[37] Zhu Y., Elbrhami A.A., Popović V., Koutchma T., Warriner K. (2019). Comparative Effects of Thermal, High Hydrostatic Pressure, and UV-C Processing on the Quality, Nutritional Attributes, and Inactivation of Escherichia Coli, Salmonella, and Listeria Introduced into Tiger Nut Milk. J. Food Prot..

[38] Zou J., Hu Y., Li K., Liu Y., Li M., Pan X., Chang X. (2023). Chestnuts in Fermented Rice Beverages Increase Metabolite Diversity and Antioxidant Activity While Reducing Cellular Oxidative Damage. Foods.

[39] Santos M.V., Banfi S., Santos R., Mota M., Raymundo A., Prista C. (2023). Improving Chestnut Physicochemical Properties through Fermentation—Development of Chestnut Amazake. Food Chem. X.

[40] Rodríguez-Solana R., Coelho N., Santos-Rufo A., Gonçalves S., Pérez-Santín E., Romano A. (2019). The Influence of In Vitro Gastrointestinal Digestion on the Chemical Composition and Antioxidant and Enzyme Inhibitory Capacities of Carob Liqueurs Obtained with Different Elaboration Techniques. Antioxidants.

[41] Goufo P., Trindade H. (2014). Rice Antioxidants: Phenolic Acids, Flavonoids, Anthocyanins, Proanthocyanidins, Tocopherols, Tocotrienols, γ-Oryzanol, and Phytic Acid. Food Sci. Nutr..

[42] Jung T.-D., Shin G.-H., Kim J.-M., Choi S.-I., Lee J.-H., Lee S.J., Park S.J., Woo K.S., Oh S.K., Lee O.-H. (2017). Comparative Analysis of γ-Oryzanol, β-Glucan, Total Phenolic Content and Antioxidant Activity in Fermented Rice Bran of Different Varieties. Nutrients.

[43] Hernández-Olivas E., Asensio-Grau A., Calvo-Lerma J., García-Hernández J., Heredia A., Andrés A. (2022). Content and Bioaccessibility of Bioactive Compounds with Potential Benefits for Macular Health in Tiger Nut Products. Food Biosci..

[44] Demarinis C., Montemurro M., Torreggiani A., Pontonio E., Verni M., Rizzello C.G. (2023). Use of Selected Lactic Acid Bacteria and Carob Flour for the Production of a High-Fibre and “Clean Label” Plant-Based Yogurt-like Product. Microorganisms.

[45] da Silva L.R., Velasco J.I., Fakhouri F.M. (2023). Use of Rice on the Development of Plant-Based Milk with Antioxidant Properties: From Raw Material to Residue. LWT.

[46] Shan L., Tyagi A., Ham H.-J., Oh D.H. (2024). Uncovering the Antiinflammatory Potential of Lactiplantibacillus Plantarum Fermented Cannabis Sativa L Seeds. npj Sci. Food.

[47] Zhao Y., Wu C., Zhu Y., Zhou C., Xiong Z., Samy Eweys A., Zhou H., Dong Y., Xiao X. (2021). Metabolomics Strategy for Revealing the Components in Fermented Barley Extracts with Lactobacillus Plantarum Dy-1. Food Res. Int..

[48] Pelegrín C.J., Ramos M., Jiménez A., Garrigós M.C. (2022). Chemical Composition and Bioactive Antioxidants Obtained by Microwave-Assisted Extraction of *Cyperus esculentus* L. By-Products: A Valorization Approach. Front. Nutr..

[49] Saeed M.M., Fernández-Ochoa Á., Saber F.R., Sayed R.H., Cádiz-Gurrea M.d.l.L., Elmotayam A.K., Leyva-Jiménez F.J., Segura-Carretero A., Nadeem R.I. (2022). The Potential Neuroprotective Effect of *Cyperus esculentus* L. Extract in Scopolamine-Induced Cognitive Impairment in Rats: Extensive Biological and Metabolomics Approaches. Molecules.

[50] Brejchova K., Balas L., Paluchova V., Brezinova M., Durand T., Kuda O. (2020). Understanding FAHFAs: From Structure to Metabolic Regulation. Prog. Lipid Res..

[51] Yore M.M., Syed I., Moraes-Vieira P.M., Zhang T., Herman M.A., Homan E.A., Patel R.T., Lee J., Chen S., Peroni O.D. (2014). Discovery of a Class of Endogenous Mammalian Lipids with Anti-Diabetic and Anti-Inflammatory Effects. Cell.

[52] Kuda O., Brezinova M., Rombaldova M., Slavikova B., Posta M., Kopecky J., Kudova E., Rossmeisl M., Flachs P. (2021). Distinct Biological Activities of Isomers from Several Families of Branched Fatty Acid Esters of Hydroxy Fatty Acids (FAHFAs). J. Lipid Res..

[53] Smit B.A., Engels W.J.M., Wouters J.T.M., Smit G. (2004). Diversity of L-Leucine Catabolism in Various Microorganisms Involved in Dairy Fermentations, and Identification of the Rate-Controlling Step in the Formation of the Potent Flavour Component 3-Methylbutanal. Appl. Microbiol. Biotechnol..

[54] Hidayat S., Yoshino K., Tokunaga C., Hara K., Matsuo M., Yonezawa K. (2003). Inhibition of Amino Acid-mTOR Signaling by a Leucine Derivative Induces G1 Arrest in Jurkat Cells. Biochem. Biophys. Res. Commun..

[55] Sumi K., Sakuda M., Munakata K., Nakamura K., Ashida K. (2021). α-Hydroxyisocaproic Acid Decreases Protein Synthesis but Attenuates TNFα/IFNγ Co-Exposure-Induced Protein Degradation and Myotube Atrophy via Suppression of iNOS and IL-6 in Murine C2C12 Myotube. Nutrients.

[56] Razola-Díaz M.d.C., Gómez-Caravaca A.M., Guerra-Hernández E.J., Garcia-Villanova B., Verardo V. (2022). New Advances in the Phenolic Composition of Tiger Nut (*Cyperus esculentus* L.) by-Products. Foods.

[57] Calderon-Montano J.M., Burgos-Moron E., Perez-Guerrero C., Lopez-Lazaro M. (2011). A Review on the Dietary Flavonoid Kaempferol. Mini Rev. Med. Chem..

[58] Pei K., Ou J., Huang J., Ou S. (2016). P-Coumaric Acid and Its Conjugates: Dietary Sources, Pharmacokinetic Properties and Biological Activities. J. Sci. Food Agric..

[59] Elfazazi K., Harrak H., Achchoub M., Benbati M. (2020). Physicochemical Criteria, Bioactive Compounds and Sensory Quality of Moroccan Traditional Carob Drink. Mater. Today Proc..

[60] Santonocito D., Granata G., Geraci C., Panico A., Siciliano E.A., Raciti G., Puglia C. (2020). Carob Seeds: Food Waste or Source of Bioactive Compounds?. Pharmaceutics.

[61] Rodríguez-Solana R., Romano A., Moreno-Rojas J.M. (2021). Carob Pulp: A Nutritional and Functional By-Product Worldwide Spread in the Formulation of Different Food Products and Beverages. A Review. Processes.

[62] Ozturk T., Ávila-Gálvez M.Á., Mercier S., Vallejo F., Bred A., Fraisse D., Morand C., Pelvan E., Monfoulet L.E., González-Sarrías A. (2024). Impact of Lactic Acid Bacteria Fermentation on (Poly)Phenolic Profile and In Vitro Antioxidant and Anti-Inflammatory Properties of Herbal Infusions. Antioxidants.

[63] Custódio L., Escapa A.L., Fernandes E., Fajardo A., Aligué R., Alberício F., Neng N., Nogueira J.M.F., Romano A. (2011). Phytochemical Profile, Antioxidant and Cytotoxic Activities of the Carob Tree (*Ceratonia siliqua* L.) Germ Flour Extracts. Plant Foods Hum. Nutr..

[64] Braune A., Gütschow M., Engst W., Blaut M. (2001). Degradation of Quercetin and Luteolin by Eubacterium Ramulus. Appl. Environ. Microbiol..

[65] Gao Y., Zhou H., Wang Y., Nussio L.G., Yang F., Ni K. (2024). Insights into Fermentation with Lactic Acid Bacteria on the Flavonoids Biotransformation of Alfalfa Silage. Chem. Biol. Technol. Agric..

[66] Zannini M., Cattivelli A., Nissen L., Conte A., Gianotti A., Tagliazucchi D. (2024). Identification, Bioaccessibility, and Antioxidant Properties of Phenolic Compounds in Carob Syrup. Foods.

[67] Ortega N., Macià A., Romero M.-P., Reguant J., Motilva M.-J. (2011). Matrix Composition Effect on the Digestibility of Carob Flour Phenols by an in-Vitro Digestion Model. Food Chem..

[68] Chait Y.A., Gunenc A., Bendali F., Hosseinian F. (2020). Simulated Gastrointestinal Digestion and in Vitro Colonic Fermentation of Carob Polyphenols: Bioaccessibility and Bioactivity. LWT.

[69] Eicher C., Coulon J., Favier M., Alexandre H., Reguant C., Grandvalet C. (2024). Citrate Metabolism in Lactic Acid Bacteria: Is There a Beneficial Effect for Oenococcus Oeni in Wine?. Front. Microbiol.

[70] Zheng Z., Wei L., Zhu M., Qian Z., Liu J., Zhang L., Xu Y. (2023). Effect of Lactic Acid Bacteria Co-Fermentation on Antioxidant Activity and Metabolomic Profiles of a Juice Made from Wolfberry and Longan. Food Res. Int..

[71] Duan W., Guan Q., Zhang H.-L., Wang F.-Z., Lu R., Li D.-M., Geng Y., Xu Z.-H. (2023). Improving Flavor, Bioactivity, and Changing Metabolic Profiles of Goji Juice by Selected Lactic Acid Bacteria Fermentation. Food Chem..

[72] van Beek S., Priest F.G. (2000). Decarboxylation of Substituted Cinnamic Acids by Lactic Acid Bacteria Isolated during Malt Whisky Fermentation. Appl. Environ. Microbiol..

[73] Fiorino G.M., Tlais A.Z.A., Losito I., Filannino P., Gobbetti M., Di Cagno R. (2023). Triacylglycerols Hydrolysis and Hydroxy- and Epoxy-Fatty Acids Release during Lactic Fermentation of Plant Matrices: An Extensive Study Showing Inter- and Intra-Species Capabilities of Lactic Acid Bacteria. Food Chem..

[74] Ciulu M., Cádiz-Gurrea M.d.l.L., Segura-Carretero A. (2018). Extraction and Analysis of Phenolic Compounds in Rice: A Review. Molecules.

[75] Sęczyk Ł., Sugier D., Świeca M., Gawlik-Dziki U. (2021). The Effect of in Vitro Digestion, Food Matrix, and Hydrothermal Treatment on the Potential Bioaccessibility of Selected Phenolic Compounds. Food Chem..

[76] Deng Y.-J., Wang S.Y. (2016). Synergistic Growth in Bacteria Depends on Substrate Complexity. J. Microbiol..

[77] Eke-Ejiofor J., Beleya E.A. (2018). Chemical and Sensory Properties of Spiced Tigernut (*Cyperus esculentus vassativa*) Drink. Int. J. Biotechnol. Food Sci..

[78] Toufeili I., Itani M., Zeidan M., Al Yamani O., Kharroubi S. (2022). Nutritional and Functional Potential of Carob Syrup Versus Date and Maple Syrups. Food Technol. Biotechnol..

[79] Duman E. (2019). Some Physico-Chemical Properties, Fatty Acid Compositions, Macro-Micro Minerals and Sterol Contents of Two Variety Tigernut Tubers and Oils Harvested from East Mediterranean Region. Food Sci. Technol..

[80] Roselló-Soto E., Barba F.J., Lorenzo J.M., Dominguez R., Pateiro M., Mañes J., Moltó J.C. (2019). Evaluating the Impact of Supercritical-CO2 Pressure on the Recovery and Quality of Oil from “Horchata” by-Products: Fatty Acid Profile, α-Tocopherol, Phenolic Compounds, and Lipid Oxidation Parameters. Food Res. Int..

[81] Youssef M.K.E., El-Manfaloty M.M., Ali H.M. (2013). Assessment of Proximate Chemical Composition, Nutritional Status, Fatty Acid Composition and Phenolic Compounds of Carob (*Ceratonia siliqua* L.). Food Public Health.

[82] Beaulieu J.C., Moreau R.A., Powell M.J., Obando-Ulloa J.M. (2022). Lipid Profiles in Preliminary Germinated Brown Rice Beverages Compared to Non-Germinated Brown and White Rice Beverages. Foods.

[83] Clemente-Villalba J., Cano-Lamadrid M., Issa-Issa H., Hurtado P., Hernández F., Carbonell-Barrachina Á.A., López-Lluch D. (2021). Comparisonon Sensory Profile, Volatile Composition and Consumer’s Acceptancefor PDO or Non-PDO Tigernut (*Cyperus esculentus* L.) Milk.. LWT.

[84] Banwo K., Taiwo O.T. (2025). Potential antioxidant activities and bioactive compounds in probiotic tiger nut date palm yogurt fermented with lactic acid bacteria. J. Food Sci. Technol..

[85] Srour N., Daroub H., Toufeili I., Olabi A. (2016). Developing a Carob-Based Milk Beverage Using Different Varieties of Carob Pods and Two Roasting Treatments and Assessing Their Effect on Quality Characteristics. J. Sci. Food Agric..

[86] Allahdad Z., Manus J., Aguilar-Uscanga B.R., Salmieri S., Millette M., Lacroix M. (2022). Physico-Chemical Properties and Sensorial Appreciation of a New Fermented Probiotic Beverage Enriched with Pea and Rice Proteins. Plant Foods Hum. Nutr..

[87] Li S., Li H., Lu L., Shao G., Guo Z., He Y., Wang Y., Yang X., Chen M., Hu X. (2024). Analysis of Rice Characteristic Volatiles and Their Influence on Rice Aroma. Curr. Res. Nutr. Food Sci..

[88] Faye T., Tamburello A., Vegarud G.E., Skeie S. (2012). Survival of Lactic Acid Bacteria from Fermented Milks in an in Vitro Digestion Model Exploiting Sequential Incubation in Human Gastric and Duodenum Juice. J. Dairy Sci..

[89] Treven P., Paveljšek D., Bogovič Matijašić B., Mohar Lorbeg P. (2024). The Effect of Food Matrix Taken with Probiotics on the Survival of Commercial Probiotics in Simulation of Gastrointestinal Digestion. Foods.

[90] Bourebaba Y., Marycz K., Mularczyk M., Bourebaba L. (2022). Postbiotics as Potential New Therapeutic Agents for Metabolic Disorders Management. Biomed. Pharmacother..

