# Supplementary material for: Modulation of Antioxidant Capacity, Nutritional Composition, Probiotic Viability After Digestion and Sensory Attributes of Plant-Based Beverages Through Lactic Acid Fermentation
Source: Foods. 2025 Apr 22;14(9):1447. doi: 10.3390/foods14091447 (PMC12071967; doi:10.3390/foods14091447)
Supplement: Supplementary file 1 [file foods-14-01447-s001.zip › foods-3574860-supplementary.pdf]

Table S1. Phytochemical compounds identified in tiger nut beverages by UHPLC-QTOF.

No.	Metabolites	RT (min)	Exact *m/z*[-]	Molecular formula	Error (ppm)	Score
1	Citric acid	1.6	191,0197	$C_6H_8O_7$	1.8	98.8
2	Homovanillic acid *	3.8	181,0506	$C_9H_{10}O_4$	1.5	95.2
3	L-leucic acid	5.6	131.0718	$C_6H_{12}O_3$	1.2	99.8
4	S-leucic acid	5.8	131.0718	$C_6H_{12}O_3$	1.2	99.8
5	Kaempferol 3’,7-diglucoside *	6.2	609.1464	$C_{27}H_{30}O_{16}$	0.6	98.5
6	4-vinylphenol *	7.0	119.0504	C_8H_8O	0.3	99.3
7	Ethyl vanillin *	7.1	165.0556	$C_9H_{10}O_3$	-0.3	98.9
8	Ferulic acid *	8.0	193.0505	$C_{10}H_{10}O_4$	0.2	99.3
9	Sinapoyl alcohol	9.3	209.0809	$C_{11}H_{14}O_4$	0.3	99.5
10	Dehydrodivanillin *	12.2	301.0714	$C_{16}H_{14}O_6$	0.3	99.0
11	Trihydroxy octadecenoic acid isomer a	15.7	329.2335	$C_{18}H_{34}O_5$	1.0	98.6
12	Trihydroxy octadecenoic acid isomer b	15.6	329.2335	$C_{18}H_{34}O_5$	1.0	98.6
13	Dihydroxyoleic acid isomer a	18.0	313.2380	$C_{18}H_{34}O_4$	-1.1	99.8
14	Dihydroxyoleic acid isomer b	18.2	313.2380	$C_{18}H_{34}O_4$	-1.1	99.8
15	Dihydroxystearic acid isomer a	18.7	315.2537	$C_{18}H_{36}O_4$	-1.4	99.6
16	Dihydroxystearic acid isomer b	19.0	315.2537	$C_{18}H_{36}O_4$	-1.4	99.6
17	Hydroxylinoleic acid isomer a	19.8	295.2282	$C_{18}H_{32}O_3$	0.9	98.8
18	Hydroxyoleic acid isomer a	20.3	297.2429	$C_9H_9NO_3$	-1.4	99.1
19	Hydroxyoleic acid isomer b	20.7	297.2432	$C_9H_9NO_3$	-0.9	99.1

* Phenolic compounds.

Table S2. Phytochemical compounds identified in carob beverages by UHPLC-QTOF.

No.	Metabolites	RT (min)	Exact *m/z*[-]	Molecular formula	Error (ppm)	Score
1	2-Dehydro-D-xylonate	1.4	163.0248	$C_5H_8O_6$	2.1	99.5
2	Citric acid	1.5	191.0197	$C_6H_8O_7$	1.8	98.8
3	2-Deoxy-D-Ribose	1.6	133.0506	$C_5H_{10}O_4$	0.9	99.9
4	(R)-2-Methylmalate	1.8	147.0299	$C_5H_8O_5$	1.4	99.8
5	2-Galloylglucose *	1.8	331.0671	$C_{13}H_{16}O_{10}$	1.6	98.9
6	Succinic acid *	1.8	117.0193	$C_4H_6O_4$	1.8	99.7
7	2-Methylcitrate	1.9	205.0534	$C_7H_{10}O_7$	1.5	98.7
8	Gallic acid *	2.3	169.0142	$C_7H_6O_5$	-1.5	98.8
9	2-O-Galloylsucrose *	2.4	493.1199	$C_{19}H_{26}O_{15}$	2.0	97.5
10	Phloroglucinol *	2.6	125.0244	$C_6H_6O_3$	0.8	99.4
11	Ethylmalonic acid	3.0	131.035	$C_5H_8O_4$	2.0	99.7
12	b-D-Xylopyranosyl-(1-4)-a-L-rhamnopyranosyl-(1-2)-L-arabinose	3.2	427.1457	$C_{16}H_{28}O_{13}$	1.3	97.9
13	a-L-Fucopyranosyl-(1-2)-b-D-galactopyranosyl-(1-2)-D-xylose	3.5	457.1563	$C_{17}H_{30}O_{14}$	1.6	99.5
14	Cynaroside A *	3.8	443.1923	$C_{21}H_{32}O_{10}$	1.4	99.6
15	3'-Methoxyfukiic acid	3.8	285.0616	$C_{12}H_{14}O_8$	1.8	97.8
16	Gallic acid 4-O-(6-galloylglucoside) *	4.1	483.078	$C_{20}H_{20}O_{14}$	1.2	97.7
17	3-propylmalic acid *	4.4	175.0612	$C_7H_{12}O_5$	1.1	99.6
18	3-O-Methylgallate *	4.6	183.0299	$C_8H_8O_5$	1.0	99.4

Table S2 cont. Phytochemical compounds identified in carob beverages by UHPLC-QTOF.

No.	Metabolites	RT (min)	Exact m/z^-	Molecular formula	Error (ppm)	Score
19	Eriocitrin *	4.7	595.1668	$C_{27}H_{32}O_{15}$	1.1	96.9
20	Gallotannin *	5.2	635.089	$C_{27}H_{24}O_{18}$	1.9	99.6
21	Gallotannin (isomer) *	5.7	635.089	$C_{27}H_{24}O_{18}$	1.9	99.6
22	Delphinidin 3-O-3",6"-O-dimalonylglucoside *	5.7	635.089	$C_{27}H_{25}O_{18}$	1.8	97.1
23	Ellagic acid +*	7.2	300.999	$C_{14}H_6O_8$	2.0	97.2
24	Myricitrin +*	7.6	463.0882	$C_{21}H_{20}O_{12}$	0.9	98.3
25	Quercetin 3-O-glucoside +*	8.1	463.0882	$C_{21}H_{20}O_{12}$	1.5	98.6
26	Benzoic acid *	8.4	121.0295	$C_7H_6O_2$	1.4	99.8
27	Quercetin 3-arabinoside +*	8.5	433.0776	$C_{20}H_{18}O_{11}$	1.7	97.9
28	Quercitrin +*	8.7	447.0933	$C_{21}H_{20}O_{11}$	2.0	98.7
29	Isochinomin	9.1	435.0933	$C_{20}H_{20}O_{11}$	2.0	98.8
30	Kaempferide 7-glucoside +*	9.2	461.1089	$C_{22}H_{22}O_{11}$	1.8	98.6
31	Phloretin 2'-O-glucuronide +*	9.5	449.1089	$C_{21}H_{22}O_{11}$	1.5	99.2
32	Apigenin 7-O-glucoside +*	10.0	431.0984	$C_{21}H_{20}O_{10}$	1.8	97.9
33	6-Hydroxykaempferol +*	10.1	301.0354	$C_{15}H_{10}O_7$	1.4	97.7
34	Eriodictyol +*	12.0	287.0561	$C_{15}H_{12}O_6$	1.6	98.2
35	Luteolin +*	12.2	285.0405	$C_{15}H_{10}O_6$	2.1	96.8
36	Quercetin +*	12.4	301.0354	$C_{15}H_{10}O_7$	0.9	98.2
37	Isorhamnetin +*	13.1	315.051	$C_{16}H_{12}O_7$	1.2	98.1
38	9S,12S,13S-trihydroxy-10E-octadecenoic acid	14.6	329.2333	$C_{18}H_{34}O_5$	1.8	97.4

Table S2 cont. Phytochemical compounds identified in carob beverages by UHPLC-QTOF.

No.	Metabolites	RT (min)	Exact m/z-	Molecular formula	Error (ppm)	Score
40	Octadecanedioic acid	17.9	313.2384	$C_{18}H_{34}O_4$	0.8	97.7
41	9,10-DiHOME	18.1	313.2384	$C_{18}H_{34}O_4$	1.7	97.1
42	L-Menthyl acetoacetate	18.3	239.1653	$C_{14}H_{24}O_3$	1.4	98.9
43	9,10-dihydroxy stearic acid	19.0	315.2541	$C_{18}H_{36}O_4$	1.8	99.0
44	Laserpitin	20.3	449.2545	$C_{25}H_{38}O_7$	1.5	98.4
45	alpha,alpha'-Trehalose 6-palmitate	20.3	579.3386	$C_{28}H_{52}O_{12}$	1.7	98.7
46	12R-hydroxy-9Z-octadecenoic acid	22.4	297.2435	$C_{18}H_{34}O_3$	1.5	98.2
47	16-hydroxy hexadecanoic acid	22.6	271.2279	$C_{16}H_{32}O_3$	0.7	99.1

* Phenolic compounds. + Metabolites identified and quantified with their authentic standards.

Table S3. Phytochemical compounds identified in rice beverages by UHPLC-QTOF.

No.	Metabolites	RT (min)	Exact *m/z*[-]	Molecular formula	Error (ppm)	Score
1	Citric acid	1.5	191.0201	$C_6H_8O_7$	1.8	98.8
2	L-leucic acid	5.6	131.0718	$C_6H_{12}O_3$	1.2	99.8
3	S-leucic acid	5.8	131.0718	$C_6H_{12}O_3$	1.2	99.8
4	p-coumaric acid *	7.0	163,0404	$C_9H_8O_3$	0.5	98.9
5	Ethyl vanillin *	7.2	165.0556	$C_9H_{10}O_3$	-0.3	99.9
6	Sinapoyl alcohol	9.5	209.0809	$C_{11}H_{14}O_4$	0.3	99.5
7	Trihydroxy octadecenoic acid isomer a	14.7	329.2335	$C_{18}H_{34}O_5$	1.0	98.6
8	Trihydroxy octadecenoic acid isomer b	14.8	329.2335	$C_{18}H_{34}O_5$	1.0	98.6
9	Dihydroxyoleic acid isomer a	18.0	313.2380	$C_{18}H_{34}O_4$	-1.1	99.8
10	Dihydroxyoleic acid isomer b	18.2	313.2380	$C_{18}H_{34}O_4$	-1.1	99.8
11	Dihydroxystearic acid isomer a	18.7	315.2537	$C_{18}H_{36}O_4$	-1.4	99.6
12	Dihydroxystearic acid isomer b	19.0	315.2537	$C_{18}H_{36}O_4$	-1.4	99.6
13	Hydroxylinoleic acid isomer a	19.8	295.2282	$C_{18}H_{32}O_3$	0.9	98.8
14	Hydroxyoleic acid isomer a	20.3	297.2429	$C_9H_9NO_3$	-1.4	99.1
15	Hydroxyoleic acid isomer b	20.7	297.2432	$C_9H_9NO_3$	-0.9	99.1

* Phenolic compounds.
